# Using a genetic/clinical risk score to stop smoking (GeTSS): randomised controlled trial

**DOI:** 10.1186/s13104-017-2831-2

**Published:** 2017-10-23

**Authors:** John A. A. Nichols, Paul Grob, Wendy Kite, Peter Williams, Simon de Lusignan

**Affiliations:** 1 Department of Clinical and Experimental Medicine, University of Surrey, Guildford, Surrey, GU2 7XH UK; 2 Jardim De Bensafrim, Lote 8, Bensafrim, 8600 069 Argave Portugal; 3 Department of Mathematics, University of Surrey, Guildford, Surrey, GU27XH UK; 4 60 Manor Way, Onslow Village, Guildford, Surrey, GU2 7RR UK

**Keywords:** Smoking cessation, Genetic testing, Lung neoplasms, Primary health care

## Abstract

**Background:**

As genetic tests become cheaper, the possibility of their widespread availability must be considered. This study involves a risk score for lung cancer in smokers that is roughly 50% genetic (50% clinical criteria). The risk score has been shown to be effective as a smoking cessation motivator in hospital recruited subjects (not actively seeking cessation services).

**Methods:**

This was an RCT set in a United Kingdom National Health Service (NHS) smoking cessation clinic. Smokers were identified from medical records. Subjects that wanted to participate were randomised to a test group that was administered a gene-based risk test and given a lung cancer risk score, or a control group where no risk score was performed. Each group had 8 weeks of weekly smoking cessation sessions involving group therapy and advice on smoking cessation pharmacotherapy and follow-up at 6 months. The primary endpoint was smoking cessation at 6 months. Secondary outcomes included ranking of the risk score and other motivators.

**Results:**

67 subjects attended the smoking cessation clinic. The 6 months quit rates were 29.4%, (10/34; 95% CI 14.1–44.7%) for the test group and 42.9% (12/28; 95% CI 24.6–61.2%) for the controls. The difference is not significant. However, the quit rate for test group subjects with a “very high” risk score was 89% (8/9; 95% CI 68.4–100%) which was significant when compared with the control group (p = 0.023) and test group subjects with moderate risk scores had a 9.5% quit rate (2/21; 95% CI 2.7–28.9%) which was significantly lower than for above moderate risk score 61.5% (8/13; 95% CI 35.5–82.3; p = 0.03).

**Conclusions:**

Only the sub-group with the highest risk score showed an increased quit rate. Controls and test group subjects with a moderate risk score were relatively unlikely to have achieved and maintained non-smoker status at 6 months.

*ClinicalTrials.gov ID* NCT01176383 (date of registration: 3 August 2010)

**Electronic supplementary material:**

The online version of this article (doi:10.1186/s13104-017-2831-2) contains supplementary material, which is available to authorized users.

## Background

Genetic tests in primary care are no longer limited by their exorbitant cost [1] leading the focus to shift from cost to the clinical value of the tests. Researchers have shown that there is a significant genetic familial component to lung cancer risk [2–6] and the recent development of gene-based tests that predict the risk of lung cancer in smokers is an example of a test which may be useful in the primary care setting [7, 8]. A research team in New Zealand has shown a correlation between a genetic test, consisting of 19 single nucleotide polymorphisms (SNPs) and one deletion mutation, and cancer risk (Additional file 1: Appendix S1). When a risk score was calculated from the results of the genetic test and clinical risk factors (COPD, family history of lung cancer and age), this was an accurate predictor for the development of lung cancers [9, 10].

**Fig. 1:**
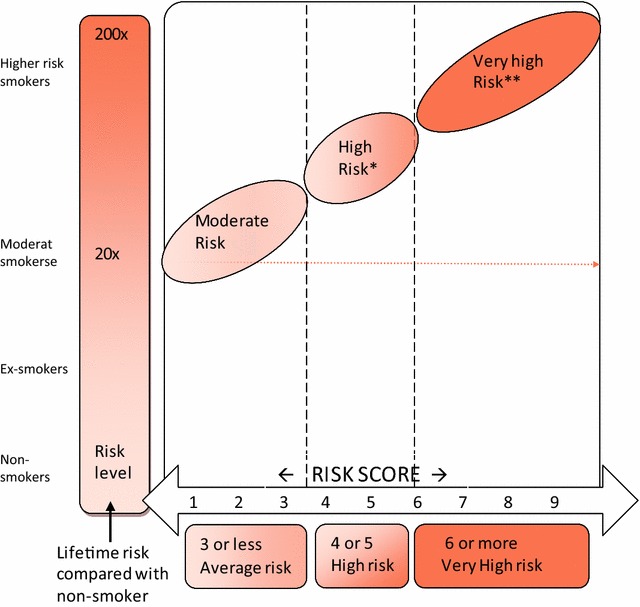
Individualised lung cancer risk score graph based on genome-wide association studies of smokers with cancer and a control group of smokers that had not developed lung cancer. Given a risk score, the level of risk and the increased risk compared with a non-smoker can be read off

They also conducted a controlled trial using this risk score for smoking cessation motivation. They recruited smokers who were recently discharged from hospital but who were not actively planning to quit smoking or enrolling in a smoking cessation programme. Follow-up was done by telephone and the lung cancer risk score was calculated and explained to test subjects using a risk graph (Fig. 1). There was a 6 month quit rate of 20% in subjects with a moderate risk score for lung cancer, 36% for high risk score and 40% for very high risk score [11, 12]. When compared with a 5% quit rate for the control group and with previous studies using telephone counselling alone [13], the absolute figures for smoking cessation with this risk score was 20–25% higher [14].

A USA hospital trial of CT screening for lung cancer in smokers and ex-smokers used the same risk score. This trial showed that subjects categorised as having a very high risk score had a 71% adherence to the CT screening protocol compared with 52% for the other two categories—high and moderate (p < 0.05). There was no evidence of demotivating effects in the moderate (lowest) risk group [15, 16]. There have, however, been no UK trials of this gene-based risk score. Our trial was designed to evaluate the risk score as a motivator in an NHS primary care smoking cessation clinic alongside the usual counselling and prescribing protocol [17].

## Methods

We published our protocol and outcome measures a priori [18]. Ethical approval was granted by Surrey Research Ethics Committee.

### Recruitment

Based on previous studies [8, 11, 12] we estimated that we needed at least 60 subjects to detect statistically significant results [18]. Subjects were recruited from a UK NHS primary care unit using computerised medical records to identify and contact smokers by post (letters signed and sent from their registered GPs). Subjects who replied stating that they wished to stop smoking by attending our clinic were randomised by the principal investigator (PI) (stratified randomisation to ensure equivalent age and gender mix) to a test group (genetic test and risk score) or control group.

### Study design and consent

Two clinics were run in parallel at the same venue but on different weekdays for test and control groups. Information sheets were posted to all subjects that had expressed an interest in attending the clinic and at first attendance, subjects were seen individually to discuss participation and they were invited to complete a consent form. An initial assessment was carried out using the Fagerström nicotine addiction score [19] and salivary cotinine [20]. Each clinic consisted of 8 smoking cessation sessions and end of trial evaluation at 6 months. A buccal swab was taken for the 20 gene test on the first or second attendance [18]. The 8 weekly smoking cessation sessions included group counselling and advice on smoking cessation pharmacotherapy (varenicline or a range of NRTs).

Lung cancer susceptibility (Fig. 1) was calculated using the Auckland formula [10]:

$$\begin{aligned} {\text{Lung cancer score}} &= \left( {\text{number of susceptible genotypes}} \right) \\ & \quad - \left( {\text{number of protective genotypes}} \right) \\ & \quad + \;3 { }\left( {\text{if lung cancer history in a first degree relative}} \right) \\ & \quad+\; 4 { }\left( {\text{if history of COPD}} \right) \\ & \quad +\;4 { }\left( {{\text{for age}} > 60 {\text{years}}} \right) \hfill \\ \end{aligned}$$

The gene-based test report included the risk score with an explanation of how the scores relate to the three different risk categories: moderate, high and very high (Fig. 1). The PI saw each patient individually (majority at smoking cessation session 3) to give a full explanation. Some swabs failed to isolate DNA and had to be repeated and some reports were slightly delayed due to administrative issues. Figure 1 also gives an estimate of how many times more likely development of lung cancer is compared with a non-smoker and from this figure the lifetime risk of cancer with continued smoking was estimated in case participants asked for this estimate.

### Study setting

The study took place in the primary care premises of a group general practitioner practice in an English suburb southwest of London. NHS primary care smoking data is very reliably recorded [20]. At the time of this study (2011–13) smoking cessation clinics were commissioned by a Surrey wide NHS body and followed national recommendations [17] which in turn followed an international consensus [17–23]. They were run by two trained NHS Surrey’s smoking cessation practitioners who were responsible for the weekly monitoring and group counselling [17].

### Primary outcome measures

The primary endpoint was smoking cessation at 6 months. Although self-reported smoking cessation is usually reliable [24], we also carried out the carbon monoxide breath test and estimation of salivary cotinine [20] at 6 months. There was no monitoring of smoking cessation in the period between the 8 week smoking cessation session and the 6 month follow-up session.

### Secondary outcome measures

Questionnaires were used to evaluate outcomes and secondary endpoints at the 8 week session and at the 6 month follow up clinic session. Although both controls and test group subjects were asked to complete the questionnaires, a question about the motivational value of a gene-based test would not have been relevant to the controls and was omitted from their version of the questionnaire. Participants who failed to attend at 8 weeks and 6 months were contacted by telephone to remind them to complete and forward their questionnaires. When participants found this too difficult, they were completed over the telephone with the PI. The questionnaires were designed to determine which subjects had quit smoking or cut down. They were also asked to score the motivators (10 for test group and 9 for controls) for their Influence in helping them to quit (Additional file 2: Appendix S2). The questions in this section were derived from, but not identical to, a previously validated questionnaire [25]. Scoring was:

- 5 = Absolute maximum
- 4 = Considerable
- 3 = Moderate
- 2 = A little
- 1 = Very little
- 0 = None
− 1 = Made me smoke more!

Scoring was calculated for individual participants as a percentage of the combined total score for all motivators for that individual. Thus, if the sum of all ten motivators is 2 + 2 + 0 + 4 + 4 + 4 + 3 + 4 + 1 + 2 = 26, then the score for a single motivator of 4 is 4/26 = 19.23%. Mean scores were calculated; selected pairs of motivators were compared using the Wilcoxon matched pairs test.

We also asked whether the participant would be likely to recommend a test for lung cancer risk to a friend or family member; an approach, although not without criticism, that is now used for evaluation of hospital and primary care across England [26]. Although this question was obviously related to the gene test in the test group the question was put to the controls in terms of an unspecified hypothetical test for risk of lung cancer. An open ended question asked participants to add comments about the concept of a lung cancer risk score.

## Results

### Patient characteristics

**Fig. 2:**
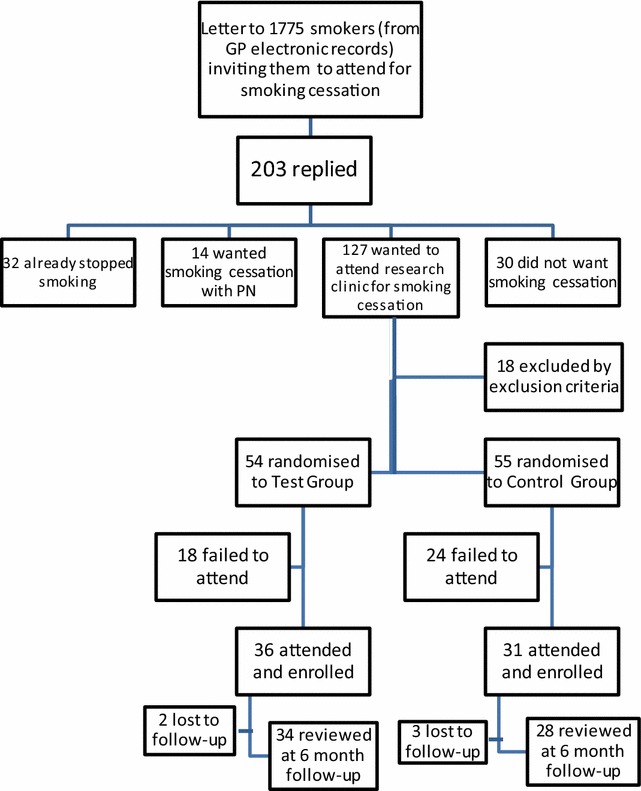
Recruitment flow chart

109 patients were randomised to test group or control group but 42 failed to attend and enrol. This was before test group recruits were offered the gene test which was not done until the first smoking cessation session. Further analysis of the 42 subjects who failed to attend and register for the trial was not possible as permission to access their records would have required attendance and completion of consent forms. 67 subjects attended and enrolled, 36 in the test group and 31 in the control group (Fig. 2). The mean age and age range were similar in both test and control groups: 49.7 (range: 23–69) years and 49.0 (range: 21–67) years in the test group and control group, respectively. Women comprised 55.6% of the test group and 54.8% of the control group. The groups did not differ significantly with respect to the years in education which was calculated based on age at which main education ceased and included tertiary education (41.7% of test group and 41.9% of controls). (unpaired *t* test: p = 0.517).

### Outcomes

#### Primary end point

For the 67 subjects enrolled in the trial 61% had quit at 8 weeks for both groups but quit rate at 6 months was: 10/34 (29.4%, 95% CI 14.1–44.7%) in the test group and 12/28 (42.9%, 95% CI 24.6–61.2%) in the control group (Table 2a). Five were completely lost to follow-up (two from the test group, three from the control group) having moved to another area. At 6 months 41.8% (28/67) failed to attend the follow-up clinic so that confirmation of smoking cessation by a negative salivary cotinine or carbon monoxide breath testing was not possible; 39 subjects did attend and their saliva and breath tests were 100% concordant with their self-reported smoking status. There were differences in nicotine addiction (see Fagerström score) and rate of completion of varenicline courses between the test group and control group (Tables 1, 2c). When the data were adjusted for these confounding factors using multiple logistic regression (Additional file 3: Appendix S3), the difference in quit rate between the control group and test group subjects with moderate/high risk scores was not significant (p = 0.076). Comparing the nine very high risk subjects with the 48 controls group subjects at 6 months, a higher proportion of the very high risk group had stopped smoking (8/9, 88.9%, 95% CI 68.4–100% versus 12/28, 42.9%, 95% CI 31.1–54.8%; Table 3, Fisher’s exact test: p = 0.023). Test group subjects with moderate risk scores had a 2/21 quit rate (9.5%; 95% CI 2.7–28.9%) which was significantly lower than for above moderate risk score of 8/13 quit rate (61.5%; 95% CI 35.5–82.3; Table 3, χ^2^ test: p = 0.03).

**Table 1:** Baseline summary of statistics for treatment groups

Demographic/smoking feature	Test group (n = 35)	Control group (n = 31)	p values (test)
Gender: female	20 (55.6%)	16 (53.3%)	0.747 (chi square)
Mean age (at start of study)	49.7	49.0	0.812 (unpaired t)
Mean age at completion of education	18.4	18.5	0.971 (unpaired t)
Years in education (excluding interruptions)	22.8	26.2	0.517 (unpaired t)
Mean pack years	32.0	28.9	0.396 (unpaired t)
Mean cigarettes/day at start	18.1	18.1	0.993 (unpaired t)
Mean Fagerström score	5.3	4.5	0.165 (unpaired t)
Mean salivary cotinine score at start	2.5	2.3	0.389 (unpaired t)
Completed course of varenicline	16 (19.4%)	19 (29%)	0.169 (chi square)

**Table 2:** Comparisons between start, 8 weeks and 6 month follow-up for attempted smoking cessation, cigarette consumption and smoking cessation therapy

Smoking cessation attempts	Currently non-smoking					
	At start	8 weeks	At 6 months follow-up			
2a						
Test group (n = 36)	0	22	10			
Control group (n = 31)	0	19	12			
Combined data for test and control groups (n = 67)	0	41	22			

Cigarette consumption	Mean values for cigarettes (or cigars × 2)/day					
	At start	8 weeks	At 6 months follow-up			
2b						
Test group (n = 36)	18.11	3.71	8.18			
Control group (n = 31)	18.10	2.29	6.68			
Combined data for test and control groups (n = 67)	18.10	3.05	7.50			

Smoking cessation therapy	Smoking cessation therapy					
	At start		8 weeks		At 6 months follow-up	
	Varenicline	NRT	Varenicline	NRT	Varenicline	NRT
2c						
Test group (n = 36)	24	12	16	14	0	NK
Control group (n = 31)	24	7	19	9	0	NK
Combined data for test and control groups (n = 67)	48	19	35	23	0	NK

*NK* not known (not recorded)

**Table 3:** Test group lung cancer risk according to result of risk score (based on genetic test and clinical criteria) and smoking cessation at 6 months

Stopped at 6 month follow-up?	Estimated risk of lung cancer			Total
	Moderate risk	High risk	Very high risk	
Stopped smoking count % who had quit smoking	2	0	8	10
	9.5%	0%	88.9%	29.4%
Still smoking count % who were still smoking	19	4	1	24
	90.5%	100.0%	11.1%	70.6%
Total count	21	4	9	34

#### Secondary end points

**Fig. 3:**
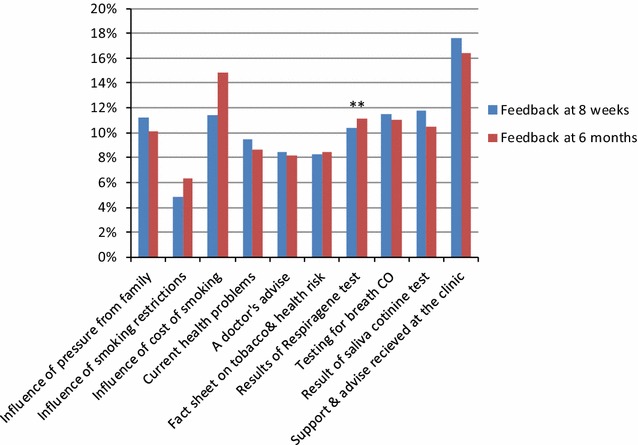
Mean values for motivators and influences that have helped to reduce or stop smoking: “Please score each of the items below according to how strong an influence they have been in helping you to quit smoking”. Scores for motivators for individual participants were calculated as percentages of the sum of total scores of the individual and mean values calculated from these percentage scores (Taken from: [31]). ** Data for “Results of Respiragene test” as a motivator is based on feedback from the test group only

The two feedback questionnaires carried out at 8 weeks and 6 months demonstrated scores for the motivating factors for subjects attempting to stop smoking (Fig. 3). Comparing the rating for the influence at 6 months of the risk score for lung cancer against each of the other motivators for the test group (the control group were not offered the genetic test and therefore not asked about its motivational value), the risk score had a significantly greater influence than smoking restrictions, current health problems, doctor’s advice, fact sheet on tobacco risk and saliva cotinine test (Wilcoxon matched pairs test: p = 0.002, 0.022, 0.007, 0.019 and 0.047 respectively); the risk score was rated as a motivator equivalent to pressure from the family, cost of smoking and carbon monoxide breath test. When comparing the other motivators against each other utilising both groups of patients, general support for smoking cessation clinic sessions was significantly more influential at 6 months than every other motivator except cost of smoking (Wilcoxon matched pairs tests: all p < 0.006; more influential than cost of smoking at 10% level: p = 0.066). There was a statistically significant higher level of confidence about recommending “a test for lung cancer” risk to family and friends amongst subjects in the test group compared with subjects in the control group at 8 weeks (Mann–Whitney U test, for friends: p = 0.003; for family: p = 0.012). This trend was less marked but still statistically significant at the 6-month follow-up (Mann–Whitney U test for friends: p = 0.033; for family: p = 0.114). There was a generally positive response to the open ended questions asking how they felt about having had the genetic test and lung cancer risk score (test group) or how they would feel about having a test that would estimate their risk of lung cancer (control group). At the 6 month follow-up 68% (95% CI 50.7–85.3%) of controls and 72% (95% CI 56.9–87.1%) of test group stated that a test for lung cancer risk would help them to cut down or quit smoking.

The open ended question also elicited a broad spectrum of opinion with several participants commenting that passive smoking and affects on the health of children and grandchildren was a very significant motivator (Table 4).

**Table 4:** Comments from patients including a representative sample of responses to question 7 at the 6 month follow-up session: test group: “How do you feel now about having had a genetic test that estimated the probability that you will develop lung cancer at some future date?”, control group: “How would you feel about having a test that estimates the probability that you will develop lung cancer at some future date if you continue smoking?”

Ref no.	Group	
	C = control T = test group	Comments
1	T	For you to use the test to personalise the message does have a big impact
2	T	The test made me stop and think. I did need help, likewise other members of group so we could help each other—empathising
3	T	My risk score was lower than expected so the “scare factor” not so effective
4	T	The Respiragene test motivated me more at the time than it does now. However, it is still good to know now
5	T	If I had realised that the course would involve sitting in a circle discussing my smoking habits and so forth, I wouldn’t have come in the first place!
6	T	If the group had carried on longer, I wouldn’t still me smoking
7	T	The test was worth having but I was mainly concerned about the affect of passive smoking on my grandchildren
8	C	The stress of separating from my wife has left me smoking again. I would like to have a risk test to help me quit again
9	C	I wouldn’t want a risk test. I believe life goes its own natural course. You’ve got to die of something
10	C	I wouldn’t mind having a test for lung cancer risk even though it is scary
11	C	I am smoking too much. I would end up worrying that the result (of a risk test) will be high but I think it’s a reality check I need
12	C	The medication (varenicline) made so much difference compared to previous attempts to quit. It took away the mental pressure
13	C	I am concerned about the affect of passive smoking on my small daughter
14	C	My orthopaedic surgeon said my fractured arm wasn’t healing due to me being a smoker

## Discussion

### Summary of principal findings

This first trial of a risk score for lung cancer with genetic and clinical components in a primary care setting showed that a very high risk score encourages smoking cessation with a quit rate 46% higher than controls (p = 0.023) but quit rates for moderate risk scores were 33.4% lower than controls. Although quit rates were identical at the end of the 8 weeks of smoking cessation sessions there was a higher lapse rate in the test group than controls. The study also demonstrated the feasibility of building genetic tests of this sort into an established smoking cessation service.

### Comparison with the literature

Our results contrast with the findings of Hopkins et al. [11, 12]. Their test group underwent gene-based testing and their control group did not whilst both groups were offered brief advice and a smoking cessation information pack with an NRT prescription. The gene-based testing used in both this study and our own study (multiple SNPs plus clinical data) is quite different from the gene-based approach in previous smoking cessation studies [8, 27]. However, in the Hopkins study, test group subjects had a better quit rate than controls regardless of the score. Possible reasons for these differences are:

1. Their hospital recruited smokers were in the precontemplative-contemplative stage whereas this study involved people from primary care who were in the action stage of quitting. Precontemplative hospital controls, without the benefit of the risk score as a motivator, may be relatively resistant to smoking behaviour change [28].
2. Their test subjects and controls were contacted by telephone after having had only the genetic test whereas our participants attended our clinic regularly for various interventions including group counselling [17]. The high score for “help from the clinic” as a motivator may have masked the motivational effect of the risk score.

Another possible explanation for poor quit rates for subjects with moderate scores is that their relatively low scores encouraged continuation of smoking. Test subjects with moderate risk scores had a 9.5% quit rate. When this was compared with all other participants this poor quit rate was significant (p = 0.022) which suggest that their relatively low risk score may have reassured them that it was safe to carry on smoking or encouraged them to lapse during the 6 month period between the 8 weekly smoking cessation sessions and the 6 month follow-up session.

## Strengths and limitations

This trial of partly genetic risk score as a smoking cessation motivator was the first to investigate a role in primary care. Only 7% of smokers expressed an interest in our smoking cessation clinic (Fig. 2) which is a fairly typical response rate [29, 30]. However, this poor recruitment means that the study is underpowered to answer some basic questions such as whether a moderate risk score reassures smokers and encourages them to continue smoking or whether a very high risk score can reliably yield such an impressive quit rate. Further research is urgently needed to clarify the significance of these findings.

Had we administered the Fagerström score questionnaire before randomisation, we could have included this value in randomisation to eliminate the level of nicotine addiction as a confounding factor.

## Conclusions

Since this study is underpowered, a larger multi-centre trial would help to clarify the risk score’s motivational value for all risk categories. However, the results suggest that this genetic test and risk estimation is acceptable to most smokers but may be more helpful to higher risk people. The risk score might be especially appropriate as a motivator for patients diagnosed with early COPD.

## Additional files

**Additional file 1: Appendix S1.** Details of genetic test (marketed as *Respiragene*).

**Additional file 2: Appendix S2.** Calculation of motivator scores.

**Additional file 3: Appendix S3.** Correction of confounding factors.

**Additional file 4: Appendix S4.** Full data set for trial.

## Abbreviations

COPD: chronic obstructive pulmonary disease
CI: confidence interval
DNA: deoxyribonucleic acid
GP: general practice
NHS: National Health Service of the United Kingdom
NRT: nicotine replacement therapy
NS: statistically non-significant
PI: principal investigator
PN: practice nurse
UK: United Kingdom
SNP: single nucleotide polymorphism
