# Supplementary material for: Using a genetic/clinical risk score to stop smoking (GeTSS): randomised controlled trial
Source: BMC Res Notes. 2017 Oct 23;10:507. doi: 10.1186/s13104-017-2831-2 (PMC5653992; doi:10.1186/s13104-017-2831-2)
Supplement: Supplementary file 1 — Additional file 1: Appendix S1. Details of genetic test (marketed as Respiragene). [file 13104_2017_2831_MOESM1_ESM.docx]

Appendix S1

**Details of the genetic test (marketed as R*espiragene*)**

The genetic test used in combination with clinical factors to calculation the lung cancer risk score (commercial name *Respiragene*) consists of 19 single nucleotide polymorphisms (SNPs) and one normal/deletion mutation. 12 SNPs involve variants that increase the risk of lung cancer (Table 1) and 7 SNP variants and the deletion mutation are protective (Table 2)

Table S1. 12 single nucleotide polymorphisms that contribute to

increased lung cancer risk

| **Risk genes** | **Alleles** | **Phenotype** | **Proposed mechanism** |
| --- | --- | --- | --- |
| a5-nAChR | A or G | AA confers susceptibility | COPD, lung damage and inflammation |
| CYP 2E1 | T or C | TT &TC confer susceptibility | Increase of Phase I detox causes build up of toxic compounds |
| Interleukin-18 | C or G | CC confers susceptibility | Excessive production of interferon |
| Interleukin-8 | A or T | TT confers susceptibility | Inhibits an anti-cancer gene |
| Interleukin 1B | A or G | GG confers susceptibility | Accelerates cell division in early cancers |
| ITGA11 | G or A | AA confers susceptibility | Upregulates growth factor IGF-1 in cancer cells |
| N-Acetylcysteine transferase | G or A | GG confers susceptibility | Increased rate of Phase I detoxification causes a build up of toxic carcinogenic compounds |
| a1-Antichymotrypsin | G or A | GG confers susceptibility | Impaired production of a protein that prevents lung damage |
| Cerberus 1 | A or G | AG&GG confer susceptibility | Dysregulation of cell growth? |
| DAT1 | G or T | GT&TT confer susceptibility | Dysregulation of dopamine signalling in cancer cells |
| TNFR1 (TNFRSF1A) | A or G | AA confers susceptibility | Impaired production of tumour necrosis factor |
| TLR9 | C or T | CC confers susceptibility | Contributes to innate inflammatory response? |

Table S2. 7 single nucleotide polymorphisms that contribute to

Reduction of lung cancer risk

| **Protective genes** | **Alleles** | **Phenotype** | **Proposed mechanism** |
| --- | --- | --- | --- |
| P73 (TP73) | C or T | CC is protective | Increases production of tumour suppressor protein P73 |
| SOD3 | C or G | GG&GC are protective | Increases production of protective antioxidant SOD3 |
| ITGB3 | A or G | GG&GA are protective | Unknown – inhibition of tumour angiogenesis? |
| DRD2 | C or Del | CDel&DelDel are protective | Beneficial alteration of dopamine signalling in cancer cells? |
| BCL2 | A or C | AA is protective | Increases apoptosis (programmed cell death) in cancer cells |
| XPD (ERCC2) | G or T | GG is protective | Reduces cancer risk by repair of damaged DNA |
| REV1 (REV1L) | C or T | CC is protective | Reduces cancer risk by repair of damaged DNA |
| FasL (TNFSF6) | C or T | TT is protective | Increases apoptosis (programmed cell death) in cancer cells |

Full details of how buccal swabs for genetic testing were taken and dispatched are included in our published protocol.^1^

The basic list of named genes was acquired from papers by Hopkins, Young et al^2,3^ and by internet searches. The SNP search engine dbSNP^4^  was especially useful

. Nichols JA, Grob P, Kite W, de Lusignan S, Williams P. Genetic test to stop smoking (GeTSS) trial protocol: randomised controlled trial of a genetic test (Respiragene) and Auckland formula to assess lung cancer risk. *BMC Pulm Med*. 2014;14:77.

2. Young RP, Hopkins RJ, Hay BA, Epton MJ, Mills GD, Black PN, Gardner HD, Sullivan R, Gamble GD. Lung cancer susceptibility model based on age, family history and genetic variants. *PLoS ONE.* 2009;4(4):e5302.

3.Young RP, Hopkins RJ, Hay BA, Gamble GD. GWAS And Candidate SNPs For COPD And Lung Cancer Combine To Identify Lung Cancer Susceptibility: Validation In A Prospective Study**.** *Am J Resp Crit Med*. 2010;181: A3738.

4. db SNP Shortgenetic variations <http://www.ncbi.nlm.nih.gov/projects/SNP/>
