# Supplementary material for: Using a genetic/clinical risk score to stop smoking (GeTSS): randomised controlled trial
Source: BMC Res Notes. 2017 Oct 23;10:507. doi: 10.1186/s13104-017-2831-2 (PMC5653992; doi:10.1186/s13104-017-2831-2)
Supplement: Supplementary file 2 — Additional file 2: Appendix S2. Calculation of motivator scores. [file 13104_2017_2831_MOESM2_ESM.docx]

Using a genetic clinical risk score to stop smoking (GeTSS): randomised controlled trial

John AA Nichols^1^*, Paul Grob^1^, Wendy Kite^2^, Peter Williams^3^ and Simon de Lusignan^1^

Appendix S2: Calculation of motivator scores

Scoring of potential motivators was recorded at 8 weeks and again at 6 months using question 4 from the feedback questionnaires. The only difference between test subjects and controls being the omission of a score for the genetic test (trade name *Respiragene*) for controls:

1. Version for Test group

1. Version for control group:

Questions were scored on a 1-5 scale with the numbers equivalent to a motivational efficacy of:

5 = Absolute maximum

4 = Considerable

3 = Moderate

2 = A little

1 = Very little

0 = None

-1= Made me smoke more!

The range of scoring varied considerably amongst the subjects. Presumably, this reflects different attitudes to form filling and to rating of the significance of motivators. The lowest range of scoring was: 0, 0, 1, 0, 0, 0, 0, 0, 2 (sum=3) and one of the highest was: 4, 0, 1, 5, 5, 3, 5, 5, 5, 5 (sum=38). Figure S1 illustrates the range of variations between these two extremes.

Figure S1. Range of total scores (sum of score for all 10 motivators)

for each of 67 participants

Total of scores for all 10 motivators per participant

Participants in order of total scores (sum of all 10 motivators)

In order to make this range of scores compatible, the scores for motivators were calculated for individual participants as a percentage of the combined total score for all motivators for that individual. Thus, if the total score for the ten motivators for an individual participant is 2+2+0+4+4+4+3+4+1+2 = 26 then a score of 4 is 4/26 = 19.23%. Mean scores for the ten motivators were calculated from this data using an Excel template (Table 1).

Figure S2 shows the data displayed as the mean values of the raw motivational score without any adjustment to compensate for the widely different variations in range of scores between individual participants. Figure S3 shows scores adjusted to compensate for variation in range of score by calculating each score as a percentage of total score of the participant as described above, then calculating mean values for this scoring system.

Figure S2. Mean of scores for motivators (without adjustment for variations in range of scoring)

Figure S3. Mean of percentages of total scores per individual participant

Wilcoxon matched pairs tests were carried out on the data shown in Figure S3 as described in the main paper to estimate the significance of differences between the degrees of influence of the various motivators.

Table S1. Example of (part of) Excel table used to calculate mean percentile values

| Columns B-K  subjects scores: | Column L  Motivator 1 | Column M  Motivator 2 | Column N  Motivator 3 | Column O  Motivator 4 | Column P  Motivator 5 | Column Q  Motivator 6 |
| --- | --- | --- | --- | --- | --- | --- |
| Subjects 44 scores | =SUM(B44:K44) | =B44/L44*100 | =(C44/L44)*100 | =(D44/L44)*100 | =(E44/L44)*100 | =(F44/L44)*100 |
| Subjects 45 scores | =SUM(B45:K45) | =B45/L45*100 | =(C45/L45)*100 | =(D45/L45)*100 | =(E45/L45)*100 | =(F45/L45)*100 |
| Subjects 46 scores | =SUM(B46:K46) | =B46/L46*100 | =(C46/L46)*100 | =(D46/L46)*100 | =(E46/L46)*100 | =(F46/L46)*100 |
| Subjects 47 scores | =SUM(B47:K47) | =B47/L47*100 | =(C47/L47)*100 | =(D47/L47)*100 | =(E47/L47)*100 | =(F47/L47)*100 |
| Subjects 48 scores | =SUM(B48:K48) | =B48/L48*100 | =(C48/L48)*100 | =(D48/L48)*100 | =(E48/L48)*100 | =(F48/L48)*100 |
| Subjects 49 scores | =SUM(B49:K49) | =B49/L49*100 | =(C49/L49)*100 | =(D49/L49)*100 | =(E49/L49)*100 | =(F49/L49)*100 |
| Subjects 50 scores | =SUM(B50:K50) | =B50/L50*100 | =(C50/L50)*100 | =(D50/L50)*100 | =(E50/L50)*100 | =(F50/L50)*100 |
| Subjects 51 scores | =SUM(B51:K51) | =B51/L51*100 | =(C51/L51)*100 | =(D51/L51)*100 | =(E51/L51)*100 | =(F51/L51)*100 |
| Subjects 52 scores | =SUM(B52:K52) | =B52/L52*100 | =(C52/L52)*100 | =(D52/L52)*100 | =(E52/L52)*100 | =(F52/L52)*100 |
| Subjects 53 scores | =SUM(B53:K53) | =B53/L53*100 | =(C53/L53)*100 | =(D53/L53)*100 | =(E53/L53)*100 | =(F53/L53)*100 |
| Subjects 54 scores | =SUM(B54:K54) | =B54/L54*100 | =(C54/L54)*100 | =(D54/L54)*100 | =(E54/L54)*100 | =(F54/L54)*100 |
| Subjects 55 scores | =SUM(B55:K55) | =B55/L55*100 | =(C55/L55)*100 | =(D55/L55)*100 | =(E55/L55)*100 | =(F55/L55)*100 |
| Subjects 56 scores | =SUM(B56:K56) | =B56/L56*100 | =(C56/L56)*100 | =(D56/L56)*100 | =(E56/L56)*100 | =(F56/L56)*100 |
| Subjects 57 scores | =SUM(B57:K57) | =B57/L57*100 | =(C57/L57)*100 | =(D57/L57)*100 | =(E57/L57)*100 | =(F57/L57)*100 |
| Subjects 58 scores | =SUM(B58:K58) | =B58/L58*100 | =(C58/L58)*100 | =(D58/L58)*100 | =(E58/L58)*100 | =(F58/L58)*100 |
| Subjects 59 scores | =SUM(B59:K59) | =B59/L59*100 | =(C59/L59)*100 | =(D59/L59)*100 | =(E59/L59)*100 | =(F59/L59)*100 |
| Subjects 60 scores | =SUM(B60:K60) | =B60/L60*100 | =(C60/L60)*100 | =(D60/L60)*100 | =(E60/L60)*100 | =(F60/L60)*100 |
| Subjects 61 scores | =SUM(B61:K61) | =B61/L61*100 | =(C61/L61)*100 | =(D61/L61)*100 | =(E61/L61)*100 | =(F61/L61)*100 |
| Subjects 62 scores | =SUM(B62:K62) | =B62/L62*100 | =(C62/L62)*100 | =(D62/L62)*100 | =(E62/L62)*100 | =(F62/L62)*100 |
| Subjects 63 scores | =SUM(B63:K63) | =B63/L63*100 | =(C63/L63)*100 | =(D63/L63)*100 | =(E63/L63)*100 | =(F63/L63)*100 |
| Subjects 64 scores | =SUM(B64:K64) | =B64/L64*100 | =(C64/L64)*100 | =(D64/L64)*100 | =(E64/L64)*100 | =(F64/L64)*100 |
| Subjects 65 scores | =SUM(B65:K65) | =B65/L65*100 | =(C65/L65)*100 | =(D65/L65)*100 | =(E65/L65)*100 | =(F65/L65)*100 |
| Subjects 66 scores | =SUM(B66:K66) | =B66/L66*100 | =(C66/L66)*100 | =(D66/L66)*100 | =(E66/L66)*100 | =(F66/L66)*100 |
| Subjects 67 scores | =SUM(B67:K67) | =B67/L67*100 | =(C67/L67)*100 | =(D67/L67)*100 | =(E67/L67)*100 | =(F67/L67)*100 |
|  |  | =AVERAGE(M2:M67) | =AVERAGE(N2:N67) | =AVERAGE(O2:O67) | =AVERAGE(P2:P67) | =AVERAGE(Q2:Q67) |

**Detailed statistics for lung cancer risk score as an effective motivator**

Comparing the rating of the influence at 6 months of the risk score against each of the other motivators for the test group, the risk score had a significantly greater influence than smoking restrictions, current health problems, Doctor’s advice, factsheet on tobacco risk and saliva cotinine test (Wilcoxon matched pairs test: p=0.002, 0.022, 0.007, 0.019 and 0.047 respectively); the genetic test and risk score were rated as a motivator equivalent to pressure from the family, cost of smoking and carbon monoxide breath test.

Although there was a statistically significant higher level of confidence about recommending a test for lung cancer risk to family and friends amongst subjects in the test group compared with subjects in the control group at 8 weeks (Mann-Whitney U test, for friends: p=0.003; for family: p=0.012) this trend was less marked but still statistically significant at the 6-month follow-up (Mann-Whitney U test for friends: p=0.033; for family: p=0.114). There was a generally positive response to the open ended questions asking how they felt about having had the gene-based test (test group) or how they would feel about having a test that would estimate their risk of lung cancer (control group). At the 6 month follow-up 68% of controls and 72% of test group stated that a test for lung cancer risk would help them to cut down or quit smoking.
