# Supplementary material for: Using a genetic/clinical risk score to stop smoking (GeTSS): randomised controlled trial
Source: BMC Res Notes. 2017 Oct 23;10:507. doi: 10.1186/s13104-017-2831-2 (PMC5653992; doi:10.1186/s13104-017-2831-2)
Supplement: Supplementary file 3 — Additional file 3: Appendix S3. Correction of confounding factors. [file 13104_2017_2831_MOESM3_ESM.docx]

Using a genetic clinical risk score to stop smoking (GeTSS): randomised controlled trial

John AA Nichols^1^*, Paul Grob^1^, Wendy Kite^2^, Peter Williams^3^ and Simon de Lusignan^1^

Appendix S2: Correction of confounding factors

There was a small bias in favour of smoking cessation for the control group due to

1. More controls were able to complete their courses of varenicline than test subjects (Figure 1)
2. Controls tended to have lower Fagerström scores for nicotine addiction (Table 1. main paper).
3. Controls tended to have a history of fewer total pack years (Table 1. Main paper).

| **Table S1.** Compare test and control groups for completion of varenicline | | |
| --- | --- | --- |
|  | Completed varenicline course Y-N | |
|  | Completed course of varenicline | Never completed a course of varenicline |
| Test group | 16 (44.4%) | 20 (55.6%) |
| Control group | 19 (61.3%) | 12 (38.7%) |

A logistic regression model was calculated on a 0 - 6 point scale based on a point scoring system to estimate the extent of confounding factors creating a bias in favour of smoking cessation in the control group. The point scoring is summarised in Table 2.

| **Table S2.** System for combined scoring participants for confounders (CSC) | | | |
| --- | --- | --- | --- |
| Fagerström score for nicotine addiction | Weakly addicted  0-3 points | Moderately addicted  (4-6 points) | Severely addicted  (7-10 points) |
| Score | 0 | 1 | 2 |
| Pack years | Light smoker  (<10 pack yrs) | Moderate smoker  (10-39.999 pack yrs) | Heavy smoker  (>40 pack yrs) |
| Score | 0 | 1 | 2 |
| Varenicline completion | Completed course | No varenicline or incomplete course of varenicline | |
| Score | 1 | 2 | |

Then inserting the combined score for confounders (CSC) alongside the lung cancer risk scores for average and very high risk as predictors of stopping at 6 months does nullify the average risk score as a predictor to the extent of making it non-significant compared with the control group (p=0.076).

The very high risk score remains significant (p=0.012) with an adjusted odds ratio of 19.597 i.e. somebody with a Respiragene "Very high risk" test result is 20 times more likely to have quit at 6 months than anybody else.

Without the CSC adjustment i.e. if the CSC is removed from the model, average risk score is also statistically significant (p=0.036) indicating that the Respiragene "Average risk" subject are less likely to give up at 6 months compared to all other groups, including controls.

So ensuring an even distribution of the CSC amongst patients allocated to Test or Control groups in future studies is important in order to maintain a level playing field. Alternatively, the Fagerström score should be calculated before randomisation so that this value could be included in randomisation to ensure an equal distribution of nicotine addiction scores between Test group and Control group.
