# Supplementary material for: Using a genetic/clinical risk score to stop smoking (GeTSS): randomised controlled trial
Source: BMC Res Notes. 2017 Oct 23;10:507. doi: 10.1186/s13104-017-2831-2 (PMC5653992; doi:10.1186/s13104-017-2831-2)
Supplement: Supplementary file 4 — Additional file 4: Appendix S4. Full data set for trial. [file 13104_2017_2831_MOESM4_ESM.pdf]

| KEY TO FULL TITLES OF COLUMNS D to CY) | |
|---|---|
| Patient code A/B (A = test group; B = control group) | abcode |
| | |
| Age at 1/10/11 **deleted** for patient confidentiality | - |
| Age band for age at 1/10/11 (5 year bands) | ageband |
| Gender | gend |
| Marital status | mstat |
| Age completed education | educn |
| Years in education (excluding interruptions) | edyrs |
| First prescription of smoking cessn. aid | prescript1 |
| Second prescription of smoking cssn. aid | prescript2 |
| Completed varenicline course Y-N | varenicomplte |
| Fagerstrom score graded low/medium/high | fagergrade |
| Pack years | packyr |
| Cigarettes (or cigarsx2)/day at start | cigs1 |
| Cigarettes (or cigarsx2)/day at 8 weeks | cigs8wk |
| Cigarettes (or cigarsx2)/day at 6 months | cigs6m |
| Cigarettes/day reduction from start of trial to 6-months follow-up clinic | cigsct6m |
| Fagerstrom score at start | fager |
| SmokeScreen cotinine test | cotinin |
| Fagerstrom + Smokescreen combined score | fagercot |
| Weekly CO breath test - 1 | breath1 |
| Weekly CO breath test - 2 | breath2 |
| Weekly CO breath test - 3 | breath3 |
| Weekly CO breath test - 4 | breath4 |
| Weekly CO breath test - 4 | breath5 |
| Weekly CO breath test - 6 | breath6 |
| Weekly CO breath test - 7 | breath7 |
| Weekly CO breath test - 8 | breath8 |
| CO breath test first reading | cofirstrecd |
| CO breath test low reader at 8 weeks | conegwk8 |
| CO breath test - 6 months | breath9 |
| Last CO reading up to and including 8 weeks | lastcoin8wk |
| Last CO breath test reading at last clinic attendance | lastco |
| CO or SmokeScreen low reader at 6 months | coneg6m |
| SmokeScreen score at 6 months | cotinin6 |
| Respiragene score | respira |
| Respiragene score genes or genes plus | geneplus |
| Gene score component of Respiragen risk score | onlygenes |
| Clinical score component of Respiragene risk score | onlyclinical |
| Percentage of respiragene risk score from genes | genecent |
| Respiragene grading | respirgr |
| Respiragene score when reach age 60 years | repirasixty |
| Repiragene grading when reach age 60 years | repirgrsixty |
| Lifetime % cancer risk if continues smoking | smkrisk |
| 5 year % cancer risk if continues as non-smoker | fiveyrquitrisk |
| Lung age from spirometry reading | lungage |
| Excess of lung age over chronological age (years) | Lungaging |
| Grading of lung health | copdgrade |
| Hypothetical upgrade if all subjects had COPD | hypotheticalcopd |

| | |
|---|---|
| New COPD awarded extra 4 risk points | copdnewfour |
| Respiragene score upgrades due to new COPD | respirupgrade |
| "Q" DENOTES VALUES FROM THE QUESTIONNAIRE ADMINISTERED AT 8 WEEKS AND 6 MONTHS | |
| Q 8 weeks:Stopped after 8 weeks course? | stop8wk |
| Q 6 months:Stopped at 6 month follow-up? | stop6m |
| Q 8 weeks:Plan to stop smoking at 8 weeks? | plan8wk |
| Q 6 months Plan to stop smoking at 8 weeks? | plan6m |
| Q 8 weeks:How many days since last cig at 8 wk? | last8wk |
| Q 6 months:How many days since last cig at 6 months? | last6m |
| Q 8 weeks:Influence of pressure from family | press8wk |
| Q 6 months:Influence of pressure from family | press6m |
| Q 8 weeks:Influence of smoking restrictions | restr8wk |
| Q 6 months:Influence of smoking restrictions | restr6m |
| Q 8 weeks:Influence of cost of smoking | cost8wk |
| Q 6 months:Influence of cost of smoking | cost6m |
| Q 8 weeks:Current health problems | health8wk |
| Q 6 months:Current health problems | health6m |
| Q 8 weeks:Doctor's advice | docadv8wk |
| Q 6 months:Doctor's advice | docadv6m |
| Q 8 weeks:Factsheet on tobacco risk | fsheet8wk |
| Q 6 months:Factsheet on tobacco risk | fsheet6m |
| Q 8 weeks:Result of respiragene test | rresult8wk |
| Q 6 months :Result of respiragene test | rresult6m |
| Q 8 weeks:Breath CO results | coresult8wk |
| Q 6 months:Breath CO results | coresult6m |
| Q 8 weeks:Saliva cotinine test | cotinine8wk |
| Q 6 months:Saliva cotinine test | cotinine6m |
| Q 8 weeks:Clinic support/advice | support8wk |
| Q 6 months:Clinic support/advice | support6m |
| Q 8 weeks:Advise friend have lung CA test? | friend8wk |
| Q 6 months:Advise friend have lung CA test? | friend6m |
| Q 8 weeks:Advise family member have lung CA test? | family8wk |
| Q 6 months:Advise family member have lung CA test? | family6m |
| How would/didyou feelabout test 8 weeks - code 1 | hyfcode1 8wk |
| Howdid/would you feel about test 8 weeks - code 2 | hyfcode2 8wk |
| How did/would you feel about test 6m - code 1 | hyfcode1 6m |
| How did/would you feel about test 6m - code 2 | hyfcode2 6m |
| Number of attendances to 8 wk course | attend8wks |
| Attended 6 month follow-up | attend6m |
| Cigarettes/day reduction from start of trial to 6-months follow-up clinic | cigscut |
| CO breathalyser difference from start to 6 months | codiff6m |
| CO breathalyser difference from start to last recorded attendance | cofirsttolast |
| CO breathalyser difference from start to 8 weeks | cofirstto8wks |
| CO breathalyser difference from start to 8 wk or last reading before 8 wk | cofirstto8lb8 |
| Respiragene score re-calculated for new COPDs | newcopdrisk |
| Hypothetical Respiragene scores if all participants had COPD | hypocopdclinic |
| Sum of motivators at 6 months | summotivatrs2 |
| Sum of motivators at 8 weeks | summotivatrs1 |
| New Respiragene score after +4 for new COPDs | newcopdscores |
| Original Respiragene score | oldrespir |

| | |
|---|---|
| Lifetime % cancer risk if continues smoking | oldlifetime |
| New COPD scores included lifetime % risk if continues smoking | newcopdlife |

| Codes for individual parameters | RESULTS → | | |
|---|---|---|---|
| A = test group; B = control group | abcode | ageband | gend |
| | B2 | 45-50 | M |
| | B3 | 20-25 | M |
| values only (no code) | B1 | 45-50 | M |
| values only (no code) | B6 | 35-40 | F |
| 1=married, 2=single, 3=divorced, 4=widowed | B8 | 55-60 | F |
| values only (no code) | B9 | 50-55 | F |
| values only (no code) | B10 | 55-60 | F |
| Codes 1-7 details on sheet 2 | B16 | 35-40 | F |
| Codes 1-5 on sheet 2 | B20 | 55-60 | M |
| 1=completed course of varenicline, 2=never completed | B24 | 20-25 | M |
| 1=low score, 2=medium score, 3=high score | B26 | 20-25 | M |
| values only (no code) | B28 | 65-70 | F |
| values only (no code) | B29 | 55-60 | F |
| values only (no code) | B32 | 50-55 | F |
| values only (no code) | B33 | 60-65 | M |
| values only (no code) | B34 | 65-70 | M |
| values only (no code) | B42 | 55-60 | F |
| values only (no code) | B44 | 45-50 | F |
| values only (no code) | B46 | 45-50 | M |
| values only (no code) | B47 | 30-35 | M |
| values only (no code) | B52 | 65-70 | M |
| values only (no code) | B25 | 60-65 | F |
| values only (no code) | A18 | 65-70 | M |
| values only (no code) | A19 | 60-65 | M |
| values only (no code) | A56 | 60-65 | M |
| values only (no code) | A58 | 50-55 | F |
| values only (no code) | A60 | 60-65 | M |
| values only (no code) | A26 | 55-60 | M |
| 0=failed to attend at 8 wk, 1=low reading, 2=smokers reading | A30 | 60-65 | F |
| values only (no code) | A55 | 40-45 | M |
| values only (no code) | B41 | 60-65 | F |
| values only (no code) | B54 | 40-45 | F |
| 0=failed to attend at 6 m, 1=low reading, 2=smokers reading | A5 | 65-70 | F |
| values only (no code) | A8 | 50-55 | F |
| values only (no code) | A46 | 45-50 | M |
| 1=gene score only, 2=gene score + clinical score | B17 | 55-60 | M |
| values only (no code) | B51 | 45-50 | M |
| values only (no code) | A7 | 65-70 | M |
| values only (no code) | A12 | 45-50 | F |
| 1-average risk, 2=high risk, 3=very high risk | B56 | 35-40 | M |
| values only (no code) | A17 | 65-70 | M |
| values only (no code) | A48 | 60-65 | F |
| values only (no code) | B45 | 55-60 | F |
| values only (no code) | A1 | 45-50 | M |
| values only (no code) | A3 | 35-40 | M |
| values only (no code) | A4 | 40-45 | M |
| Codes 0-4 details on sheet 2 | A11 | 50-55 | F |
| values only (no code) | A13 | 35-40 | F |

| | | | |
|---|---|---|---|
| values only (no code) | A21 | 45-50 | F |
| Codes 0-4 details on sheet 2 | A23 | 30-35 | M |
| | A28 | 55-60 | F |
| 0=lost to follow-up, 1=yes, 2=no | A33 | 40-45 | F |
| 0=lost to follow-up, 1=yes, 2=no | A34 | 50-55 | F |
| 0=stopped, 1=yes, 2=n0, 3=don't know | A38 | 35-40 | F |
| 0=stopped, 1=yes, 2=n0, 3=don't know | A39 | 35-40 | F |
| values only (no code) | A43 | 45-50 | M |
| values only (no code) | A44 | 30-35 | F |
| Codes -1 to 5 details on sheet 2 | A50 | 30-35 | F |
| Codes -1 to 5 details on sheet 2 | A51 | 55-60 | F |
| Codes -1 to 5 details on sheet 2 | A54 | 50-55 | F |
| Codes -1 to 5 details on sheet 2 | A57 | 30-35 | M |
| Codes -1 to 5 details on sheet 2 | A59 | 55-60 | F |
| Codes -1 to 5 details on sheet 2 | A61 | 45-50 | F |
| Codes -1 to 5 details on sheet 2 | A62 | 20-25 | M |
| Codes -1 to 5 details on sheet 2 | B19 | 50-55 | M |
| Codes -1 to 5 details on sheet 2 | B23 | 40-45 | F |
| Codes -1 to 5 details on sheet 2 | B43 | 45-50 | F |
| Codes -1 to 5 details on sheet 2 | | | |
| Codes -1 to 5 details on sheet 2 | | | |
| Codes -1 to 5 details on sheet 2 | | | |
| Codes -1 to 5 details on sheet 2 | | | |
| Codes -1 to 5 details on sheet 2 | | | |
| Codes -1 to 5 details on sheet 2 | | | |
| Codes -1 to 5 details on sheet 2 | | | |
| Codes -1 to 5 details on sheet 2 | | | |
| Codes -1 to 5 details on sheet 2 | | | |
| Codes -1 to 5 details on sheet 2 | | | |
| 0=no entry, 1=yes, 2=no, 3=unsure | | | |
| 0=no entry, 1=yes, 2=no, 3=unsure | | | |
| 0=no entry, 1=yes, 2=no, 3=unsure | | | |
| 0=no entry, 1=yes, 2=no, 3=unsure | | | |
| Codes 0-6 details on sheet 2 | | | |
| Codes 0-6 details on sheet 2 | | | |
| Codes 0-6 details on sheet 2 | | | |
| Codes 0-6 details on sheet 2 | | | |
| values only (no code) | | | |
| 0=failed to attend, 1=attended | | | |
| values only (no code) | | | |
| values only (no code) | | | |
| values only (no code) | | | |
| values only (no code) | | | |
| values only (no code) | | | |
| values only (no code) | | | |
| values only (no code) | | | |
| values only (no code) | | | |
| values only (no code) | | | |
| values only (no code) | | | |
| values only (no code) | | | |

values only (no code)
values only (no code)

| mstat | educn | edyrs | prescript1 | prescript2 | varenicom | fagergrade | packyr | cigs1 |
|---|---|---|---|---|---|---|---|---|
| 3 | 17 | 17 | 1 | 0 | 1 | 2 | 30 | 40 |
| 2 | 16 | 16 | 1 | 0 | 1 | 1 | 1 | 12 |
| 2 | 18 | 18 | 1 | 0 | 1 | 1 | 10 | 20 |
| 1 | 25 | 25 | 3 | 0 | 2 | 0 | 6 | 10 |
| 3 | 11 | 11 | 6 | 0 | 2 | 2 | 23 | 10 |
| 1 | 16 | 16 | 1 | 0 | 1 | 1 | 32 | 20 |
| 1 | 16 | 16 | 1 | 0 | 1 | 0 | 25 | 10 |
| 2 | 19 | 19 | 1 | 0 | 1 | 2 | 9 | 15 |
| 1 | 15 | 15 | 2 | 4 | 2 | 0 | 44 | 22 |
| 2 | 16 | 16 | 1 | 0 | 1 | 0 | 14 | 10 |
| 2 | 17 | 17 | 1 | 0 | 1 | 0 | 5 | 15 |
| 1 | 21 | 21 | 4 | 0 | 2 | 0 | 33 | 20 |
| 3 | 15 | 15 | 4 | 0 | 2 | 0 | 29 | 20 |
| 1 | 16 | 16 | 1 | 0 | 1 | 0 | 55 | 15 |
| 1 | 49 | 20 | 2 | 0 | 2 | 0 | 24 | 10 |
| 1 | 17 | 17 | 1 | 0 | 1 | 1 | 14 | 10 |
| 1 | 16 | 16 | 1 | 0 | 1 | 0 | 36 | 15 |
| 1 | 21 | 21 | 8 | 0 | 2 | 0 | 26 | 15 |
| 2 | 19 | 19 | 1 | 0 | 1 | 1 | 53 | 30 |
| 2 | 18 | 18 | 1 | 0 | 1 | 1 | 13 | 20 |
| 1 | 15 | 15 | 1 | 0 | 1 | 1 | 32 | 20 |
| 3 | 16 | 16 | 1 | 0 | 1 | 0 | 55 | 20 |
| 1 | 15 | 15 | 2 | 4 | 2 | 2 | 41 | 20 |
| 1 | 16 | 16 | 1 | 0 | 1 | 2 | 68 | 30 |
| 1 | 15 | 15 | 1 | 0 | 1 | 0 | 47 | 20 |
| 3 | 16 | 16 | 6 | 0 | 2 | 1 | 35 | 20 |
| 1 | 16 | 16 | 1 | 0 | 1 | 1 | 54 | 10 |
| 1 | 19 | 19 | 1 | 0 | 1 | 2 | 41 | 20 |
| 1 | 16 | 16 | 1 | 0 | 1 | 0 | 21 | 15 |
| 3 | 21 | 21 | 1 | 0 | 1 | 0 | 19 | 10 |
| 3 | 17 | 17 | 1 | 0 | 1 | 1 | 43 | 20 |
| 1 | 17 | 17 | 6 | 0 | 2 | 1 | 60 | 40 |
| 1 | 19 | 19 | 1 | 0 | 1 | 1 | 50 | 15 |
| 3 | 40 | 26 | 2 | 4 | 2 | 2 | 26 | 15 |
| 1 | 16 | 16 | 1 | 0 | 1 | 2 | 39 | 25 |
| 2 | 15 | 15 | 1 | 0 | 1 | 2 | 45 | 15 |
| 2 | 16 | 16 | 6 | 0 | 2 | 2 | 37 | 20 |
| 1 | 19 | 19 | 1 | 0 | 1 | 1 | 54 | 12 |
| 3 | 19 | 19 | 6 | 0 | 2 | 2 | 39 | 25 |
| 2 | 16 | 16 | 3 | 4 | 2 | 2 | 38 | 30 |
| 1 | 16 | 16 | 1 | 0 | 1 | 0 | 48 | 10 |
| 1 | 21 | 21 | 6 | 0 | 2 | 0 | 22 | 15 |
| 3 | 15 | 15 | 6 | 0 | 2 | 1 | 39 | 17 |
| 1 | 18 | 18 | 1 | 0 | 1 | 0 | 32 | 12 |
| 1 | 21 | 21 | 2 | 0 | 2 | 0 | 9 | 10 |
| 1 | 16 | 16 | 2 | 0 | 2 | 2 | 27 | 20 |
| 1 | 16 | 16 | 3 | 0 | 2 | 1 | 35 | 20 |
| 2 | 28 | 28 | 7 | 0 | 2 | 2 | 20 | 20 |

| | | | | | | | | |
|---|---|---|---|---|---|---|---|---|
| 2 | 19 | 19 | 1 | 0 | 1 | 1 | 18 | 15 |
| 1 | 20 | 20 | 3 | 0 | 2 | 2 | 20 | 20 |
| 4 | 16 | 16 | 3 | 0 | 2 | 1 | 30 | 15 |
| 1 | 16 | 16 | 6 | 0 | 2 | 1 | 27 | 15 |
| 3 | 15 | 15 | 1 | 0 | 1 | 1 | 38 | 18 |
| 1 | | 19 | 1 | 0 | 1 | 2 | 22 | 20 |
| 2 | 18 | 18 | 3 | 0 | 2 | 1 | 14 | 15 |
| 1 | 15 | 15 | 6 | 0 | 2 | 2 | 41 | 25 |
| 2 | 18 | 18 | 6 | 0 | 2 | 2 | 14 | 10 |
| 2 | 16 | 16 | 6 | 0 | 2 | 1 | 31 | 25 |
| 1 | 17 | 17 | 4 | 0 | 2 | 0 | 44 | 20 |
| 1 | 16 | 16 | 6 | 0 | 2 | 2 | 34 | 20 |
| 2 | 16 | 16 | 1 | 0 | 1 | 0 | 20 | 20 |
| 1 | 24 | 24 | 6 | 0 | 2 | 1 | 31 | 20 |
| 2 | 18 | 18 | 8 | 0 | 2 | 1 | 38 | 30 |
| 2 | 17 | 17 | 1 | 0 | 1 | 2 | 8 | 20 |
| 1 | 22 | 22 | 1 | 0 | 1 | 0 | 7 | 10 |
| 1 | 29 | 29 | 2 | 0 | 2 | 0 | 21 | 10 |
| 3 | 16 | 16 | 1 | 0 | 1 | 2 | 40 | 20 |

| cigs8wk | cigs6m | cigsct6m | fager | cotinin | fagercot | breath1 | breath2 | breath3 |
|---|---|---|---|---|---|---|---|---|
| 10 | 25 | 15 | 7 | 3 | 10 | 57 | 56 | 50 |
| 0 | 0 | 12 | 5 | 1 | 6 |  | 12 |  |
| 0 | 0 | 20 | 4 |  |  |  | 12 | 9 |
| 0 | 0 | 10 | 1 | 1 | 2 | 9 |  |  |
| 0 | 0 | 10 | 8 | 3 | 11 | 17 |  | 3 |
| 0 | 0 | 20 | 5 | 3 | 8 | 44 | 25 | 11 |
| 0 | 7 | 3 | 3 | 1 | 4 | 7 | 11 | 4 |
| 0 | 0 | 15 | 7 | 2 | 9 | 30 | 22 |  |
| 2 | 0 | 22 | 2 | 3 | 5 | 21 | 21 | 17 |
| 3 | 0 | 10 | 3 | 2 | 5 | 14 | 24 | 21 |
| 0 |  |  | 2 | 2 | 4 | 25 | 16 | 10 |
| 0 | 0 | 20 | 3 | 3 | 6 |  | 14 | 15 |
| 5 | 0 | 20 | 9 | 2 | 11 | 26 |  |  |
| 0 | 0 | 15 | 6 | 2 | 8 | 44 | 34 | 32 |
| 0 | 6 | 4 | 1 | 1 | 2 | 20 | 19 |  |
| 0 | 4 | 6 | 4 | 3 | 7 | 19 | 2 |  |
| 0 | 0 | 15 | 3 | 2 | 5 | 40 | 23 | 28 |
| 6 | 6 | 9 | 1 | 3 | 4 |  |  |  |
| 0 |  |  | 6 | 2 | 8 | 9 | 24 | 3 |
| 0 |  |  | 5 | 2 | 7 | 34 | 20 |  |
| 3 | 5 | 15 | 4 | 2 | 6 | 25 | 20 | 12 |
| 0 | 3 | 17 | 2 | 3 | 5 | 39 | 25 | 4 |
| 12 | 10 | 10 | 7 | 3 | 10 | 31 | 24 |  |
| 0 | 0 | 30 | 7 | 2 | 9 | 17 | 18 | 3 |
| 0 | 0 | 20 | 5 | 3 | 8 | 15 | 17 | 15 |
| 0 | 0 | 20 | 5 | 3 | 8 | 17 | 28 | 3 |
| 0 | 0 | 10 | 4 | 3 | 7 | 19 | 10 | 13 |
| 0 | 15 | 5 | 8 | 1 | 9 | 11 | 6 | 6 |
| 0 | 3 | 12 | 2 | 3 | 5 | 19 |  | 15 |
| 0 | 1 | 9 | 3 | 3 | 6 | 9 | 3 | 2 |
| 0 | 2 | 18 | 5 | 2 | 7 | 35 | 23 | 20 |
| 18 | 38 | 2 | 6 | 3 | 9 | 23 | 18 | 17 |
| 0 | 0 | 15 | 5 | 3 | 8 | 11 | 11 | 7 |
| 3 | 8 | 7 | 7 | 3 | 10 | 18 |  |  |
| 0 | 10 | 15 | 7 | 3 | 10 | 22 | 26 | 19 |
| 0 | 15 | 0 | 7 | 3 | 10 | 14 | 14 | 16 |
| 1 | 6 | 14 | 8 | 3 | 11 | 30 |  |  |
| 0 | 0 | 12 | 5 | 2 | 7 | 21 |  | 5 |
| 0 | 0 | 25 | 7 | 3 | 10 | 45 | 49 | 4 |
| 0 | 30 | 0 | 8 | 3 | 11 | 49 | 47 | 50 |
| 0 | 0 | 10 | 1 | 1 | 2 | 6 | 4 | 11 |
| 10 | 15 | 0 | 3 | 2 | 5 | 21 | 16 |  |
| 13 | 15 | 2 | 4 | 2 | 6 | 13 |  | 11 |
| 0 | 0 | 12 | 1 | 2 | 3 | 24 | 12 | 2 |
| 2 | 5 | 5 | 2 | 2 | 4 | 27 | 30 | 26 |
| 0 | 20 | 0 | 4 | 3 | 7 | 19 | 21 | 2 |
| 20 | 20 | 0 | 4 |  |  | 33 |  |  |
| 1 | 15 | 5 | 8 | 2 | 10 | 33 | 32 | 32 |

| | | | | | | | | |
|---|---|---|---|---|---|---|---|---|
| 0 | 12 | 3 | 4 | 1 | 5 | 25 | 25 | 20 |
| 0 | 20 | 0 | 8 | 2 | 10 | 28 | 31 | 28 |
| 4 | 10 | 5 | 5 | 2 | 7 | 21 | 25 | 24 |
| 5 | 10 | 5 | 4 | 2 | 6 | 36 | 30 | 4 |
| 0 | | | 6 | 3 | 9 | 36 | 34 | 13 |
| 0 | 12 | 8 | 7 | 2 | 9 | 41 | 31 | 29 |
| 5 | 6 | 9 | 6 | 3 | 9 | | | |
| 20 | 13 | 12 | 9 | 3 | 12 | 22 | 15 | 2 |
| 0 | | | 7 | 3 | 10 | 20 | 2 | 2 |
| 12 | 10 | 15 | 5 | 3 | 8 | 40 | | 3 |
| 0 | 10 | 10 | 3 | 3 | 5 | 9 | 5 | 3 |
| 0 | 0 | 20 | 9 | 3 | 12 | 29 | | |
| 0 | 18 | 2 | 7 | 1 | 4 | 15 | 28 | 20 |
| 10 | 5 | 15 | 4 | 3 | 7 | 15 | 12 | |
| 30 | 20 | 10 | 6 | 3 | 9 | 36 | 29 | |
| 0 | 10 | 10 | 7 | 3 | 10 | | 8 | 32 |
| 0 | 3 | 7 | 0 | 2 | 2 | 22 | | 3 |
| 0 | 12 | -2 | 2 | 3 | 5 | 6 | 8 | |
| 10 | 10 | 10 | 9 | 3 | 12 | 36 | | 23 |

| breath4 | breath5 | breath6 | breath7 | breath8 | cofirstrecd | conegwk8 | breath9 | lastcoin8wl |
|---|---|---|---|---|---|---|---|---|
| 35 | | 36 | | 29 | 57 | 2 | | 29 |
| | | 1 | | | 12 | 0 | | 1 |
| | 2 | | 2 | 1 | 12 | 1 | 4 | 1 |
| | | | | | 9 | 0 | 5 | 9 |
| | 2 | 1 | 1 | 1 | 17 | 1 | 5 | 1 |
| 2 | 2 | | | 1 | 44 | 1 | 2 | 2 |
| 2 | | | | | 7 | 0 | | 2 |
| | | | | | 30 | 0 | | 22 |
| | 20 | | | 18 | 21 | 2 | | 18 |
| | | | 12 | | 14 | 0 | 4 | 12 |
| | 4 | | 4 | 5 | 25 | 1 | | 5 |
| 13 | 6 | 4 | | 2 | 14 | 1 | | 2 |
| | | | | | 26 | 0 | 3 | 26 |
| 2 | 4 | 2 | 2 | 1 | 44 | 1 | 3 | 8 |
| | | | | | 20 | | | 19 |
| | | | | | 19 | | | 2 |
| | 1 | 2 | 2 | 3 | 40 | 1 | 1 | 1 |
| | | | | | | 0 | | 4 |
| 1 | | | 3 | 4 | 9 | 1 | | 4 |
| 5 | 2 | | 2 | 4 | 34 | 1 | | 4 |
| 9 | | 11 | 24 | | 25 | 0 | | 24 |
| 2 | 1 | 1 | | 1 | 39 | 1 | 6 | 1 |
| | | | | | 31 | 0 | | 24 |
| 2 | 2 | | | 2 | 17 | 1 | 4 | 2 |
| | 5 | 3 | 6 | | 15 | 1 | | 6 |
| 2 | | | | | 17 | 0 | 2 | 2 |
| 1 | 2 | 2 | 1 | 1 | 19 | 1 | 2 | 1 |
| | 7 | 4 | | 3 | 11 | 1 | 29 | 3 |
| 13 | 2 | 1 | 1 | 1 | 19 | 1 | 8 | 1 |
| | 1 | | 2 | 3 | 9 | 1 | | 3 |
| | 0 | | 5 | 1 | 35 | 1 | 6 | 1 |
| | | 17 | 19 | 12 | 23 | 2 | 1 | 12 |
| 6 | | 1 | | 1 | 11 | 1 | | 1 |
| | | | | | 18 | 0 | | 18 |
| 1 | 2 | 4 | | 2 | 22 | 1 | | 2 |
| 3 | 2 | 2 | 3 | 1 | 14 | 1 | 14 | 1 |
| 14 | 8 | | | | 30 | 0 | 14 | 8 |
| 4 | | 3 | 4 | 3 | 21 | 1 | | 3 |
| 2 | | 2 | | 3 | 45 | 1 | 4 | 3 |
| 3 | 1 | 1 | | | 49 | 0 | | 1 |
| 3 | 2 | 2 | 3 | 2 | 6 | 1 | 13 | 2 |
| | | | | | | 0 | | 16 |
| | | | | | 13 | 0 | | 11 |
| 2 | 4 | 2 | 2 | 2 | 24 | 1 | 5 | 2 |
| 12 | 7 | | | | 27 | 0 | | 7 |
| 2 | 4 | 3 | 2 | 5 | 19 | 1 | 25 | 5 |
| | | | | | 33 | 0 | | 33 |
| | 1 | | 2 | 4 | 33 | 1 | | 4 |

| | | | | | | | | |
|---|---|---|---|---|---|---|---|---|
| 3 | | 4 | 4 | | 25 | 0 | 16 | 4 |
| 31 | 7 | 2 | | | 28 | 0 | | 2 |
| | | | | | 21 | 0 | | 24 |
| 9 | 11 | | 9 | 7 | 36 | 2 | 26 | 7 |
| 2 | 1 | | | 3 | 36 | 1 | | 3 |
| 2 | 2 | 3 | 3 | 1 | 41 | 1 | 25 | 1 |
| | | | | | | 0 | | 9 |
| | | | | | 22 | 0 | 10 | 2 |
| 2 | 5 | 3 | 4 | 3 | 20 | 1 | | 3 |
| 1 | | | | | 40 | 0 | | 1 |
| 3 | 2 | 7 | 2 | 3 | 9 | 1 | 15 | 3 |
| | | | | | 29 | 0 | | 29 |
| 15 | 2 | 7 | 1 | 2 | 15 | 1 | | 2 |
| | | | | | 15 | 0 | | 12 |
| | | | | | 36 | 0 | | 29 |
| 15 | 4 | 4 | 1 | 3 | 8 | 1 | | 3 |
| 2 | 3 | 2 | | 2 | 22 | 1 | | 2 |
| | | | | | 6 | 0 | 19 | 8 |
| 9 | 8 | | | | 36 | 0 | 14 | 8 |

| lastco | coneg6m | cotinin6 | respira | geneplus | onlygenes | onlyclinical | genecent | respirgr |
|---|---|---|---|---|---|---|---|---|
| 29 | 0 | | | | | | | |
| 1 | 0 | | | | | | | |
| 4 | 1 | | | | | | | |
| 5 | 1 | | | | | | | |
| 5 | 1 | | | | | | | |
| 2 | 1 | | | | | | | |
| 2 | 0 | | | | | | | |
| 22 | 0 | | | | | | | |
| 18 | 0 | | | | | | | |
| 4 | 1 | 0 | | | | | | |
| 5 | 0 | | | | | | | |
| 2 | 1 | 0 | | | | | | |
| 3 | 1 | 0 | | | | | | |
| 3 | 1 | 0 | | | | | | |
| 19 | 0 | | | | | | | |
| 2 | 0 | | | | | | | |
| 1 | 1 | 0 | | | | | | |
| 4 | 0 | | | | | | | |
| 4 | 0 | | | | | | | |
| 4 | 0 | | | | | | | |
| 24 | 0 | | | | | | | |
| 6 | 2 | 1 | 11 | 2 | 1 | 10 | 9.1 | 3 |
| 24 | 2 | 1 | 9 | 2 | 1 | 8 | 11.1 | 3 |
| 4 | 1 | 0 | 7 | 2 | 1 | 6 | 14.3 | 3 |
| 6 | 1 | 0 | 7 | 2 | 1 | 6 | 14.3 | 3 |
| 2 | 1 | 0 | 6 | 2 | 1 | 5 | 16.7 | 3 |
| 2 | 1 | 0 | 9 | 2 | 2 | 7 | 22.2 | 3 |
| 29 | 2 | 2 | 4 | 2 | 1 | 3 | 25 | 2 |
| 8 | 2 | 3 | 4 | 2 | 1 | 3 | 25 | 2 |
| 3 | 0 | | 4 | 2 | 1 | 3 | 25 | 2 |
| 6 | 2 | 1 | 8 | 2 | 2 | 6 | 25 | 3 |
| 1 | 2 | 1 | 4 | 2 | 1 | 3 | 25 | 2 |
| 1 | 0 | 0 | 10 | 2 | 3 | 7 | 30 | 3 |
| 18 | 0 | | 3 | 2 | 1 | 2 | 33.3 | 1 |
| 2 | 2 | | 3 | 1 | 1 | 2 | 33.3 | 1 |
| 14 | 2 | 3 | 6 | 2 | 2 | 4 | 33.3 | 3 |
| 14 | 2 | 1 | 5 | 2 | 2 | 3 | 40 | 2 |
| 3 | 1 | 0 | 7 | 2 | 3 | 4 | 42.9 | 3 |
| 4 | 1 | 0 | 7 | 2 | 3 | 4 | 42.9 | 3 |
| 1 | 2 | 3 | 7 | 2 | 3 | 4 | 42.9 | 3 |
| 13 | 1 | 0 | 8 | 2 | 4 | 4 | 50 | 3 |
| 16 | 2 | | 2 | 2 | 1 | 1 | 50 | 1 |
| 11 | 2 | 1 | 6 | 2 | 3 | 3 | 50 | 3 |
| 5 | 1 | 0 | 2 | 1 | 2 | 0 | 100 | 1 |
| 0 | 0 | | 1 | 1 | 1 | 0 | 100 | 1 |
| 25 | 2 | | 2 | 1 | 2 | 0 | 100 | 1 |
| 33 | 0 | | 1 | 1 | 1 | 0 | 100 | 1 |
| 4 | 2 | 1 | 4 | 1 | 4 | 0 | 100 | 2 |

| | | | | | | | | |
|---|---|---|---|---|---|---|---|---|
| 16 | 2 | 2 | 1 | 1 | 1 | 0 | 100 | 1 |
| 2 | 0 | | 1 | 1 | 1 | 0 | 100 | 1 |
| 24 | 0 | | 1 | 1 | 1 | 0 | 100 | 1 |
| 26 | 2 | 3 | 3 | 1 | 3 | 0 | 100 | 1 |
| 3 | 0 | | 1 | 1 | 1 | 0 | 100 | 1 |
| 25 | 2 | 2 | 2 | 1 | 2 | 0 | 100 | 1 |
| 9 | 2 | | 1 | 1 | 1 | 0 | 100 | 1 |
| 10 | 2 | 3 | 1 | 1 | 1 | 0 | 100 | 1 |
| 3 | 0 | | 1 | 1 | 1 | 0 | 100 | 1 |
| 1 | 0 | | 1 | 1 | 1 | 0 | 100 | 1 |
| 15 | 2 | 2 | 2 | 1 | 2 | 0 | 100 | 1 |
| 29 | 0 | | 2 | 1 | 2 | 0 | 100 | 1 |
| 2 | 0 | | 1 | 1 | 1 | 0 | 100 | 1 |
| 12 | 0 | | 1 | 1 | 1 | 0 | 100 | 1 |
| 29 | 0 | | 3 | 1 | 3 | 0 | 100 | 1 |
| 3 | 0 | | 1 | 1 | 1 | 0 | 100 | 1 |
| 2 | 0 | | 1 | 2 | 1 | 0 | 100 | 1 |
| 19 | 2 | 2 | 4 | 1 | 4 | 0 | 100 | 2 |
| 14 | 2 | 3 | 1 | 1 | 1 | 0 | 100 | 1 |

| repirasixty | repirgrsixty | smkrisk | fiveyrquitri | lungage | Lungaging | copdgrade | hypothetic | copdnewfo |
|---|---|---|---|---|---|---|---|---|
| | | | | | | 0 | 4 | 0 |
| | | | | | | 0 | 4 | 0 |
| | | | | 84 | 37 | 3 | 4 | 4 |
| | | | | 37 | 0 | 3 | 4 | 4 |
| | | | | 59 | 0 | 2 | 4 | 0 |
| | | | | 55 | 5 | 1 | 4 | 0 |
| | | | | | | 0 | 4 | 0 |
| | | | | | 20 | 4 | 0 | 0 |
| | | | | | | 0 | 4 | 0 |
| | | | | 20 | 0 | 1 | 4 | 0 |
| | | | | | | 0 | 4 | 0 |
| | | | | 66 | 0 | 1 | 4 | 0 |
| | | | | 50 | 0 | 1 | 4 | 0 |
| | | | | 71 | 18 | 3 | 4 | 4 |
| | | | | | | 0 | 4 | 0 |
| | | | | | | 0 | 4 | 0 |
| | | | | 57 | 0 | 1 | 4 | 0 |
| | | | | | | 0 | 4 | 0 |
| | | | | | | 0 | 4 | 0 |
| | | | | | | 0 | 4 | 0 |
| | | | | | | 0 | 4 | 0 |
| 11 | 3 | 60 | 5 | 70 | 8 | 4 | 0 | 0 |
| 9 | 3 | 50 | 7 | | 20 | 4 | 0 | 0 |
| 7 | 3 | 50 | 6 | | 12 | 4 | 0 | 0 |
| 7 | 3 | 50 | 3 | 86 | 24 | 3 | 4 | 4 |
| 10 | 3 | 50 | 3 | 51 | 0 | 3 | 4 | 4 |
| 9 | 3 | 50 | 3 | 74 | 10 | 1 | 4 | 0 |
| 8 | 3 | 47 | 4 | | 32 | 3 | 4 | 4 |
| 4 | 2 | 15 | 8 | 75 | 0 | 1 | 4 | 0 |
| 8 | 3 | 40 | 0.5 | | | 0 | 4 | 0 |
| 8 | 3 | 60 | 5 | 91 | 30 | 3 | 4 | 4 |
| 8 | 3 | 40 | 0.5 | 59 | 19 | 4 | 0 | 0 |
| 10 | 3 | 50 | 15 | 94 | 35 | 4 | 0 | 0 |
| 7 | 3 | 37 | 3.5 | | 0 | 1 | 4 | 0 |
| 7 | 3 | 44 | 0.5 | | 11 | 1 | 4 | 0 |
| 10 | 3 | 55 | 3 | 78 | | 0 | 4 | 0 |
| 9 | 3 | 43 | 3 | 59 | 10 | 4 | 0 | 0 |
| 7 | 3 | 50 | 10 | | 19 | 3 | 4 | 4 |
| 11 | 3 | 54 | 3 | | 8 | 2 | 4 | 0 |
| 11 | 3 | 60 | 0.5 | 52 | 13 | 3 | 4 | 4 |
| 8 | 3 | 50 | 10 | 75 | 10 | 3 | 4 | 4 |
| 2 | 1 | 15 | 6 | | 16 | 3 | 4 | 4 |
| 10 | 3 | 60 | 2 | 62 | 6 | 2 | 4 | 0 |
| 6 | 3 | 30 | 3.5 | | 18 | 2 | 4 | 0 |
| 4 | 2 | 19 | 0.5 | | | 0 | 4 | 0 |
| 6 | 3 | 23 | 2 | | 9 | 2 | 4 | 0 |
| 4 | 2 | 24 | 3 | | | 0 | 4 | 0 |
| 8 | 3 | 50 | 1 | | 18 | 2 | 4 | 0 |

| | | | | | | | | |
|---|---|---|---|---|---|---|---|---|
| 4 | 2 | 22 | 1 | | 0 | 1 | 4 | 0 |
| 4 | 2 | 19 | 0.5 | | | 0 | 4 | 0 |
| 4 | 2 | 15 | 7 | | | 0 | 4 | 0 |
| 7 | 3 | 31 | 1 | | 6 | 2 | 4 | 0 |
| 4 | 2 | 14 | 2 | | | 0 | 4 | 0 |
| 6 | 3 | 29 | 0.5 | 69 | 31 | 3 | 4 | 4 |
| 4 | 2 | 18 | 0.5 | 38 | 0 | 1 | 4 | 0 |
| 5 | 2 | 27 | 2 | 58 | 13 | 1 | 4 | 0 |
| 5 | 2 | 22 | 0.5 | | | 0 | 4 | 0 |
| 3 | 1 | 16 | 0.5 | | | 0 | 4 | 0 |
| 6 | 3 | 38 | 3 | 62 | 4 | 1 | 4 | 0 |
| 6 | 3 | 35 | 3 | | | 3 | 4 | 4 |
| 4 | 2 | 17 | 0.5 | | | 0 | 4 | 0 |
| 4 | 2 | 32 | 1.5 | | | 0 | 4 | 0 |
| 7 | 3 | 43 | 3 | | | 0 | 4 | 0 |
| 4 | 2 | 18 | 0.5 | | | 0 | 4 | 0 |
| 4 | 2 | 32 | 1.5 | | 41 | 4 | 0 | 0 |
| 8 | 3 | 40 | 0.5 | 32 | 0 | 1 | 4 | 0 |
| 4 | 2 | 19 | 0.5 | 45 | 0 | 1 | 4 | 0 |

| respirupgra | stop8wk | stop6m | plan8wk | plan6m | last8wk | last6m | press8wk | press6m |
|---|---|---|---|---|---|---|---|---|
| 0 | 2 | 2 | 1 | 1 | 1 | 0.1 | 0 | 0 |
| 0 | 1 | 1 | 0 | 0 | 60 | 225 | 4 | 2 |
| 0 | 1 | 1 | 0 | 0 | 18 | 200 | 5 | 4 |
| 0 | 1 | 1 | 0 | 0 | 30 | 210 | 2 | 3 |
| 0 | 1 | 1 | 0 | 0 | 32 | 240 | 5 | 4 |
| 0 | 1 | 1 | 0 | 0 | 28 | 200 | 1 | -1 |
| 0 | 1 | 2 | 0 | 1 | 35 | 1 | 3 | 2 |
| 0 | 1 | 1 | 0 | 0 | 45 | 154 | 4 | 5 |
| 0 | 2 | 1 | 1 | 0 | 0.1 | 110 | 0 | 0 |
| 0 | 2 | 1 | 1 | 0 | 2 | 56 | 1 | 5 |
| 0 | 1 | 0 | 1 |  | 2 |  | 4 |  |
| 0 | 1 | 1 | 0 | 0 | 11 | 210 | 1 | 0 |
| 0 | 2 | 1 | 1 | 0 | 0.1 | 100 | 0 | 0 |
| 0 | 1 | 1 | 0 | 0 | 28 | 170 | 3 | 3 |
| 0 | 1 | 2 | 0 | 1 | 50 | 0.2 | 4 | 0 |
| 0 | 1 | 2 | 0 | 1 | 28 | 1 | 2 | 1 |
| 0 | 1 | 1 | 0 | 0 | 25 | 30.2 | 5 | 3 |
| 0 | 2 | 2 | 1 | 1 | 2 | 0.3 | 4 | 5 |
| 0 | 2 | 0 | 1 |  | 2 |  | 0 |  |
| 0 | 1 | 0 | 0 |  | 21 |  | 2 |  |
| 0 | 2 | 2 | 0 | 1 | 1 | 0.2 | 1 | 4 |
| 1 | 1 | 2 | 0 | 1 | 28 | 3 | 4 | 4 |
| 1 | 2 | 2 | 1 | 0 | 0.1 | 0.1 | 4 | 1 |
| 1 | 1 | 1 | 0 | 0 | 36 | 90 | 3 | 4 |
| 4 | 1 | 1 | 0 | 0 | 18 | 25 | 2 | 3 |
| 4 | 1 | 1 | 0 |  | 42 | 2 | 5 | 4 |
| 1 | 1 | 1 | 0 | 0 | 7 | 28 | 4 | 3 |
| 3 | 1 | 2 | 0 | 1 | 39 | 0.1 | 3 | 3 |
| 1 | 1 | 2 | 0 | 1 | 15 | 0.5 | 3 | 4 |
| 1 | 1 | 2 | 0 | 1 | 5 | 0.5 | 3 | 3 |
| 4 | 2 | 2 | 1 | 1 | 3 | 0.3 | 5 | 0 |
| 1 | 2 | 2 | 1 | 1 | 0.5 | 0.1 | 4 | 4 |
| 1 | 1 | 1 | 0 | 0 | 8 | 21 | 4 | 5 |
| 1 | 2 | 2 | 1 | 3 | 0.2 | 0.1 | 2 | 0 |
| 1 | 1 | 2 | 0 | 1 | 30 | 0.1 | 0 | 0 |
| 1 | 1 | 2 | 0 | 1 | 28 | 1 | 4 | 5 |
| 1 | 2 | 2 | 1 | 1 | 0.5 | 0.2 | 0 | 1 |
| 4 | 1 | 1 | 0 | 0 | 31 | 200 | 2 | 0 |
| 4 | 1 | 1 | 0 | 0 | 30 | 200 | 3 | 4 |
| 4 | 1 | 2 | 0 | 1 | 44 | 0.1 | 3 | 0 |
| 4 | 1 | 1 | 0 | 0 | 27 | 30.5 | 3 | 3 |
| 2 | 2 | 2 | 1 | 1 | 0.1 | 0.1 | 5 | 0 |
| 1 | 2 | 2 | 1 | 3 | 0.1 | 0.1 | 1 | 2 |
| 1 | 1 | 1 | 0 | 0 | 35 | 350 | 5 | 5 |
| 1 | 2 | 2 | 1 | 0 | 2 | 0.1 | 5 | 5 |
| 1 | 1 | 2 | 0 | 1 | 31 | 0.1 | 3 | 3 |
| 1 | 2 | 2 | 1 | 3 | 0.1 | 0.1 | 2 | 1 |
| 1 | 2 | 2 | 1 | 1 | 1 | 0.1 | 5 | 0 |

| | | | | | | | | |
|---|---|---|---|---|---|---|---|---|
| 1 | 1 | 2 | 0 | 1 | 15 | 0.1 | -1 | -1 |
| 1 | 2 | 2 | 1 | 1 | 0.1 | 0.1 | 5 | 5 |
| 1 | 2 | 2 | 1 | 1 | 0.5 | 0.1 | 4 | 3 |
| 1 | 2 | 2 | 1 | 1 | 0.1 | 0.1 | 0 | 3 |
| 1 | 1 | 0 | 0 | | 32 | | 0 | |
| 2 | 1 | 2 | 0 | 1 | 22 | 0.1 | 5 | 4 |
| 1 | 2 | 2 | 1 | 1 | 0.2 | 0.2 | 2 | 0 |
| 1 | 2 | 2 | 1 | 3 | 0 | 0.1 | 0 | 3 |
| 1 | 1 | 0 | 0 | | 37 | | 1 | |
| 1 | 2 | 2 | 1 | 1 | 0.1 | 0.1 | 3 | 5 |
| 1 | 1 | 2 | 0 | 1 | 35 | 0.1 | 3 | 2 |
| 2 | 1 | 1 | 0 | 0 | 35 | 17 | 5 | 4 |
| 1 | 1 | 2 | 0 | 1 | 18 | 0.1 | 2 | 3 |
| 2 | 2 | 2 | 1 | | 0.1 | 0.2 | -1 | 5 |
| 1 | 2 | 2 | 2 | 1 | | 0.1 | | -1 |
| 1 | 1 | 2 | 0 | 1 | 15 | 0.2 | 5 | 4 |
| 2 | 1 | 2 | 0 | 1 | 32 | 2 | 0 | 1 |
| 1 | 1 | 2 | 0 | 1 | 42 | 0.1 | 0 | 0 |
| 1 | 2 | 2 | 1 | 1 | 0.1 | 0.2 | 0 | 4 |

| restr8wk | restr6m | cost8wk | cost6m | health8wk | health6m | docadv8wk | docadv6m | fsheet8wk |
|---|---|---|---|---|---|---|---|---|
| 0 | 2 | 4 | 4 | 0 | 0 | 0 | 2 | 3 |
| 0 | 2 | 2 | 3 | 0 | 2 | 0 | 1 | 4 |
| 0 | 0 | 1 | 2 | 1 | 2 | 2 | 2 | 2 |
| 1 | 4 | 1 | 1 | 1 | 1 | 0 | 1 | 0 |
| 0 | 0 | 0 | 4 | 4 | 0 | 4 | 4 | 4 |
| 0 | 0 | 4 | 4 | 4 | 4 | 3 | 0 | 2 |
| 0 | 0 | 3 | 5 | 3 | 0 | 3 | 2 | 2 |
| 3 | 0 | 0 | 1 | 2 | 4 | 3 | 1 | 4 |
| 1 | 0 | 0 | 0 | 0 | 0 | 0 | 1 | 0 |
| 2 | 1 | 2 | 5 | 0 | 0 | 0 | 5 | 2 |
| 2 |  | 3 |  | 1 |  | 2 |  | 2 |
| 0 | 3 | 1 | 3 | 3 | 5 | 4 | 3 | 2 |
| 0 | 0 | 0 | 1 | 5 | 0 | 2 | 0 | 0 |
| 3 | 3 | 4 | 5 | 1 | 3 | 3 | 2 | 2 |
| 0 | 0 | 4 | 0 | 5 | 5 | 3 | 0 | 2 |
| 2 | 0 | 2 | 1 | 5 | 5 | 5 | 4 | 5 |
| 3 | 1 | 4 | 3 | 3 | 0 | 3 | 3 | 3 |
| 0 | 3 | 4 | 4 | 0 | 0 | 2 | 2 | 0 |
| 2 |  | 0 |  | 0 |  | 0 |  | 1 |
| 0 |  | 5 |  | 0 |  | 0 |  | 0 |
| 5 | 0 | 5 | 5 | 5 | 0 | 1 | 1 | 4 |
| 4 | 4 | 4 | 4 | 4 | 2 | 3 | 2 | 4 |
| 2 | 0 | 3 | 3 | 4 | 5 | 4 | 4 | 2 |
| 0 | 1 | 0 | 5 | 0 | 1 | 5 | 5 | 0 |
| 0 | 0 | 1 | 0 | 3 | 0 | 0 | 0 | 0 |
| 0 | 0 | 5 | 2 | 2 | 4 | 5 | 2 | 2 |
| 3 | 2 | 2 | 2 | 4 | 0 | 2 | 3 | 2 |
| 0 | 1 | 2 | 3 | 1 | 0 | 1 | 3 | 2 |
| 2 | 4 | 2 | 2 | 1 | 3 | 1 | 3 | 2 |
| 1 | 0 | 3 | 2 | 3 | 5 | 2 | 2 | 4 |
| 4 | 4 | 5 | 5 | 5 | 4 | 1 | 0 | 2 |
| 0 | 0 | 2 | 2 | 5 | 0 | 4 | 2 | 4 |
| 0 | 0 | 1 | 1 | 5 | 3 | 5 | 4 | 3 |
| 2 | 3 | 0 | 1 | 3 | 1 | 2 | 1 | 3 |
| 0 | 0 | 3 | 4 | 0 | 4 | 0 | 3 | 2 |
| 0 | 1 | 4 | 3 | 5 | 4 | 5 | 5 | 4 |
| 0 | 1 | 2 | 4 | 5 | 3 | 4 | 3 | 4 |
| 0 | 3 | 5 | 5 | 0 | 0 | 2 | 2 | 2 |
| 0 | 2 | 0 | 0 | 2 | 2 | 0 | 0 | 2 |
| 2 | 2 | 5 | 4 | 3 | 0 | 3 | 0 | 2 |
| 0 | 3 | 1 | 1 | 5 | 2 | 5 | 3 | 3 |
| 0 | 0 | 0 | 0 | 0 | 0 | 0 | 0 | 2 |
| 1 | 3 | 4 | 4 | 0 | 3 | 1 | 3 | 1 |
| 1 | 2 | 0 | 1 | 0 | 0 | 1 | 2 | 1 |
| 4 | 4 | 1 | 4 | 1 | 1 | 4 | 4 | 3 |
| 0 | 1 | 1 | 1 | 2 | 3 | 1 | 4 | 0 |
| 2 | 0 | 4 | 3 | 1 | 0 | 1 | 0 | 1 |
| 5 | 1 | 5 | 5 | 5 | 0 | 5 | 0 | 5 |

| | | | | | | | | |
|---|---|---|---|---|---|---|---|---|
| 3 | 5 | 3 | 4 | 3 | 3 | 2 | 4 | 2 |
| 0 | 1 | 4 | 4 | 0 | 0 | 3 | 1 | 0 |
| 1 | 3 | 4 | 4 | 5 | 4 | 4 | 4 | 4 |
| 5 | 5 | 4 | 4 | 3 | 1 | 2 | 1 | 3 |
| 5 | | 2 | | 0 | | 5 | | 5 |
| 1 | 1 | 4 | 4 | 3 | 3 | 3 | 1 | 1 |
| 0 | 0 | 5 | 4 | 0 | 4 | 1 | 0 | 0 |
| 0 | 0 | 5 | 4 | 0 | 3 | 3 | 3 | 2 |
| -1 | | 0 | | 3 | | 0 | | 0 |
| 1 | 0 | 5 | 5 | 1 | 0 | 3 | 1 | 3 |
| 0 | 0 | 3 | 4 | 4 | 4 | 1 | 0 | 1 |
| 0 | 0 | 0 | 0 | 0 | 5 | 0 | 2 | 0 |
| 0 | 1 | 2 | 5 | 0 | 1 | 0 | 2 | 4 |
| 3 | 0 | 4 | 3 | 3 | 3 | 0 | 0 | 4 |
| | 5 | | 3 | | 4 | | 0 | |
| 5 | 3 | 4 | 4 | 2 | 0 | 0 | 3 | 0 |
| 3 | 2 | 4 | 3 | 1 | 0 | 0 | 0 | 2 |
| 0 | 3 | 2 | 5 | 2 | 0 | 0 | 1 | 0 |
| 0 | 1 | 3 | 4 | 4 | 3 | 4 | 5 | 1 |

| fsheet6m | rresult8wk | rresult6m | coresult8w | coresult6m | cotinine8w | cotinine6m | support8w | support6m |
|---|---|---|---|---|---|---|---|---|
| 3 | | | 2 | 0 | 2 | 0 | 4 | 5 |
| 3 | | | 3 | 4 | 2 | 4 | 5 | 4 |
| 0 | | | 2 | 2 | 0 | 0 | 3 | 4 |
| 2 | | | 1 | 1 | 1 | 1 | 3 | 3 |
| 0 | | | 5 | 5 | 3 | 4 | 5 | 4 |
| 0 | | | 2 | 0 | 2 | 0 | 4 | 4 |
| 3 | | | 4 | 4 | 4 | 4 | 5 | 4 |
| 2 | | | 4 | 4 | 3 | 4 | 5 | 4 |
| 0 | | | 0 | 0 | 4 | 0 | 4 | 4 |
| 4 | | | 3 | 4 | 0 | 0 | 1 | 2 |
| | | | 4 | | 3 | | 4 | |
| 1 | | | 2 | 0 | 2 | 0 | 2 | 3 |
| 0 | | | 0 | 0 | 1 | 0 | 0 | 2 |
| 4 | | | 4 | 4 | 3 | 3 | 3 | 3 |
| 1 | | | 2 | 3 | 4 | 3 | 4 | 4 |
| 0 | | | 5 | 3 | 5 | 2 | 5 | 2 |
| 4 | | | 4 | 4 | 4 | 3 | 4 | 4 |
| 0 | | | 0 | 0 | 5 | 4 | 1 | 0 |
| | | | 4 | | 3 | | 5 | |
| | | | 0 | | 0 | | 4 | |
| 0 | | | 3 | 4 | 5 | 2 | 4 | 4 |
| 4 | | | 4 | 4 | 2 | 2 | 5 | 5 |
| 2 | 4 | 5 | 3 | 3 | 4 | 3 | 3 | 3 |
| 5 | 0 | 5 | 0 | 5 | 0 | 5 | 5 | 5 |
| 4 | 0 | 3 | 0 | 3 | 0 | 3 | 4 | 3 |
| 0 | 5 | 5 | 5 | 3 | 4 | 4 | 5 | 5 |
| 2 | 2 | 2 | 3 | 2 | 3 | 2 | 4 | 5 |
| 2 | 3 | 3 | 2 | 3 | 4 | 3 | 4 | 4 |
| 3 | 3 | 3 | 3 | 3 | 3 | 3 | 4 | 4 |
| 2 | 2 | 4 | 1 | 2 | 1 | 3 | 4 | 4 |
| 3 | | | 4 | 4 | 4 | 4 | 5 | 4 |
| 4 | | | 4 | 4 | 4 | 4 | 4 | 4 |
| 3 | 5 | 5 | 5 | 3 | 5 | 4 | 5 | 4 |
| 3 | 2 | 1 | 2 | 1 | 2 | 1 | 0 | 1 |
| 3 | 4 | 4 | 3 | 4 | 2 | 3 | 4 | 1 |
| 0 | | | 4 | 0 | 2 | 4 | 5 | 5 |
| 4 | | | 3 | 4 | 5 | 4 | 2 | 4 |
| 1 | 2 | 3 | 2 | 3 | 2 | 3 | 3 | 4 |
| 0 | 3 | 3 | 2 | 2 | 2 | 2 | 3 | 4 |
| 2 | | | 2 | 4 | 3 | 2 | 5 | 5 |
| 1 | 3 | 2 | 5 | 2 | 3 | 2 | 4 | 4 |
| 0 | 4 | 0 | 2 | 4 | 4 | 5 | 3 | 2 |
| 3 | | | 1 | 3 | 3 | 3 | 4 | 4 |
| 0 | 1 | 2 | 1 | 2 | 1 | 2 | 4 | 4 |
| 1 | 5 | 1 | 5 | 1 | 5 | 1 | 4 | 4 |
| 3 | 4 | 4 | 4 | 4 | 4 | 3 | 5 | 5 |
| 0 | 1 | 2 | 1 | 1 | 1 | 1 | 2 | 4 |
| 3 | 5 | 4 | 5 | 3 | 5 | 4 | 5 | 4 |

| | | | | | | | | |
|---|---|---|---|---|---|---|---|---|
| 3 | 2 | 3 | 2 | 3 | 1 | 3 | 5 | 5 |
| 2 | 1 | 3 | 4 | 4 | 1 | 3 | 5 | 1 |
| 2 | 4 | 3 | 4 | 2 | 4 | 2 | 2 | -1 |
| 3 | 0 | 3 | 4 | 3 | 0 | 3 | 4 | 4 |
| | 5 | | 5 | | 5 | | 5 | |
| 4 | 2 | 1 | 1 | 4 | 1 | 1 | 5 | 1 |
| 1 | 3 | 0 | 0 | 0 | 4 | 0 | 2 | 0 |
| 3 | 2 | 3 | 4 | 3 | 4 | 3 | 4 | 3 |
| | 1 | | 4 | | 0 | | 4 | |
| 2 | 3 | 3 | 4 | 3 | 4 | 2 | 4 | 4 |
| 0 | 1 | 2 | 1 | 3 | 1 | 3 | 4 | 5 |
| 0 | 4 | 3 | 3 | 2 | 3 | 2 | 3 | 2 |
| 3 | 3 | 0 | 4 | 3 | 4 | 0 | 5 | 5 |
| 2 | 0 | 3 | 3 | 3 | 4 | 3 | 3 | 4 |
| 4 | | 4 | | 4 | | 4 | | 0 |
| 4 | 3 | 4 | 3 | 4 | 3 | 4 | 5 | 5 |
| 1 | | | 3 | 4 | 2 | 4 | 3 | 4 |
| 3 | | | 2 | 2 | 4 | 3 | 3 | 4 |
| 4 | | | 4 | 4 | 4 | 4 | 5 | 4 |

| friend8wk | family6m | family8wk | family6m | hyfcode1 8wk | hyfcode2 8wk | hyfcode1 8wk |
|---|---|---|---|---|---|---|
| 2 | 1 | 2 | 1 | 3 | 6 | 1 |
| 1 | 1 | 1 | 1 | 1 | 1 | 6 |
| 1 | 3 | 1 | 3 | 4 | 3 | 6 |
| 1 | 3 | 1 | 1 | 2 | 1 | 2 |
| 1 | 1 | 1 | 1 | 1 | 1 | 2 |
| 1 | 1 | 1 | 1 | 1 | 1 | 0 |
| 3 | 3 | 3 | 3 | 4 | 5 | 4 |
| 1 | 3 | 1 | 1 | 2 | 1 | 2 |
| 0 | 2 | 0 | 2 | 0 | 0 | 2 |
| 2 | 3 | 1 | 1 | 4 | 5 | 2 |
| 1 |  | 1 |  | 1 | 1 | 0 |
| 3 | 3 | 3 | 3 | 4 | 5 | 4 |
| 1 | 3 | 1 | 1 | 2 | 1 | 2 |
| 3 | 1 | 3 | 1 | 3 | 6 | 2 |
| 2 | 3 | 2 | 3 | 4 | 5 | 3 |
| 3 | 3 | 3 | 3 | 4 | 5 | 4 |
| 1 | 1 | 1 | 1 | 1 | 1 | 2 |
| 3 | 3 | 3 | 3 | 1 | 3 | 1 |
| 1 |  | 1 |  | 2 | 1 | 0 |
| 3 |  | 1 |  | 3 | 6 | 0 |
| 1 | 1 | 1 | 1 | 2 | 1 | 6 |
| 3 | 1 | 3 | 1 | 1 | 4 | 1 |
| 1 | 1 | 1 | 1 | 2 | 1 | 2 |
| 1 | 1 | 1 | 1 | 6 | 4 | 3 |
| 1 | 1 | 1 | 1 | 1 | 1 | 2 |
| 1 | 1 | 1 | 1 | 2 | 1 | 2 |
| 1 | 1 | 1 | 1 | 1 | 1 | 1 |
| 1 | 3 | 1 | 3 | 1 | 4 | 2 |
| 2 | 1 | 2 |  | 0 | 0 | 1 |
| 1 | 1 | 1 | 1 | 2 | 1 | 2 |
| 3 | 1 | 3 | 1 | 4 | 5 | 2 |
| 1 | 1 | 1 | 1 | 1 | 1 | 1 |
| 1 | 1 | 1 | 1 | 2 | 1 | 2 |
| 3 | 3 | 3 | 3 | 5 | 1 | 5 |
| 1 | 1 | 1 | 1 | 2 | 1 | 1 |
| 1 | 1 | 1 | 1 | 1 | 1 | 1 |
| 1 | 1 | 1 | 1 | 2 | 1 | 2 |
| 1 | 1 | 1 | 1 | 3 | 2 | 1 |
| 1 | 3 | 1 | 1 | 2 | 1 | 2 |
| 1 | 3 | 1 | 3 | 1 | 1 | 2 |
| 1 | 1 | 1 | 1 | 1 | 1 | 2 |
| 1 | 1 | 1 | 1 | 2 | 1 | 1 |
| 1 | 1 | 1 | 1 | 2 | 1 | 2 |
| 1 | 1 | 1 | 1 | 1 | 2 | 4 |
| 1 | 1 | 1 | 1 | 2 | 1 | 0 |
| 1 | 1 | 1 | 1 | 1 | 1 | 2 |
| 1 | 1 | 1 | 1 | 2 | 1 | 2 |
| 1 | 1 | 1 | 1 | 1 | 3 | 2 |

| | | | | | | |
|---|---|---|---|---|---|---|
| 1 | 1 | 1 | 1 | 1 | 1 | 1 |
| 1 | 1 | 1 | 1 | 5 | 1 | 5 |
| 1 | 3 | 1 | 3 | 0 | 0 | 0 |
| 1 | 3 | 1 | 3 | 0 | 0 | 1 |
| 1 | | 1 | | 1 | 1 | 0 |
| 1 | 1 | 1 | 1 | 2 | 1 | 1 |
| 1 | 1 | 1 | 1 | 2 | 1 | 5 |
| 1 | 1 | 1 | 1 | 2 | 1 | 5 |
| 1 | | 1 | | 1 | 6 | 0 |
| 1 | 1 | 1 | 1 | 5 | 1 | 2 |
| 1 | 1 | 1 | 1 | 2 | 1 | 2 |
| 1 | 1 | 1 | 1 | 2 | 1 | 2 |
| 1 | 1 | 1 | 1 | 0 | 0 | 3 |
| 1 | 1 | 1 | 1 | 2 | 1 | 2 |
| | 3 | | 1 | 2 | 1 | 1 |
| 1 | | 1 | | 2 | 1 | 1 |
| 1 | 1 | 1 | 1 | 2 | 1 | 1 |
| 1 | 1 | 1 | 1 | 2 | 1 | 2 |
| 1 | 1 | 1 | 1 | 2 | 1 | 2 |

| hyfcode2 6m | attend8wk | attend6m | commentcode | commentco | cigscut | codiff6m | cofirsttolas |
|---|---|---|---|---|---|---|---|
| 1 | 5 | 0 | 10 | 5 | 15 | | 28 |
| 2 | 2 | 0 | | 9 | 12 | | 11 |
| 2 | 5 | 1 | 0 | 0 | 20 | | 8 |
| 1 | 1 | 1 | 0 | 0 | 10 | 4 | 4 |
| 1 | 7 | 1 | 0 | 0 | 10 | 12 | 12 |
| 0 | 7 | 1 | 0 | 0 | 20 | 42 | 42 |
| 5 | 4 | 0 | 2 | 8 | 3 | | 5 |
| 1 | 2 | 0 | 8 | 1 | 15 | | 8 |
| 2 | 5 | 1 | 7 | | 22 | | 3 |
| 1 | 5 | 1 | 0 | 0 | 10 | 10 | 10 |
| | 6 | 0 | 1 | | | | 20 |
| 5 | 7 | 0 | | 5 | 20 | | 12 |
| 1 | 1 | 1 | 6 | 2 | 20 | 23 | 23 |
| 1 | 8 | 1 | 9 | | 15 | 41 | 41 |
| 6 | 2 | 0 | 4 | | 4 | | 1 |
| 5 | 2 | 0 | 6 | 5 | 6 | | 17 |
| 1 | 7 | 1 | 0 | 0 | 15 | 39 | 39 |
| 1 | 1 | 0 | 4 | | 9 | | |
| | 6 | 0 | 1 | | | | 5 |
| | 6 | 0 | 1 | | | | 30 |
| 2 | 6 | 0 | 9 | | | | 1 |
| 1 | 7 | 1 | | 6 | 17 | 33 | 33 |
| 1 | 2 | 0 | 9 | | 10 | | 7 |
| 4 | 6 | 1 | | 8 | 30 | 13 | 13 |
| 1 | 6 | 1 | 9 | | 20 | | 9 |
| 1 | 4 | 1 | | 4 | 20 | 15 | 15 |
| 1 | 8 | 1 | 8 | | 10 | 17 | 17 |
| 1 | 6 | 1 | 0 | 0 | 5 | -18 | -18 |
| 1 | 7 | 1 | 10 | | 12 | 11 | 11 |
| 1 | 6 | 0 | | 8 | 9 | | 6 |
| 1 | 7 | 1 | | 3 | 18 | 29 | 29 |
| 1 | 6 | 0 | 9 | 6 | 2 | 22 | 22 |
| 1 | 6 | 0 | 8 | | 15 | | 10 |
| 2 | 1 | 0 | 5 | | 7 | | 0 |
| 1 | 7 | 0 | 0 | 0 | 15 | | 20 |
| 1 | 8 | 1 | | 6 | -5 | 0 | 0 |
| 1 | 3 | 1 | | 3 | 14 | 16 | 16 |
| 1 | 6 | 0 | 2 | | 12 | | 18 |
| 1 | 6 | 1 | 0 | 0 | 25 | 41 | 41 |
| 1 | 3 | 0 | 4 | 6 | | | 48 |
| 1 | 8 | 1 | 0 | 0 | 10 | -7 | -7 |
| 2 | 2 | 0 | 3 | | | | 5 |
| 1 | 2 | 0 | | 8 | 2 | | 2 |
| 2 | 8 | 1 | | | 12 | 19 | 19 |
| 0 | 5 | 0 | 2 | | 5 | | 27 |
| 1 | 8 | 1 | | 3 | 0 | -6 | -6 |
| 1 | 1 | 0 | 5 | | 0 | | 0 |
| 1 | 6 | 0 | 7 | 1 | 5 | | 29 |

| | | | | | | | |
|---|---|---|---|---|---|---|---|
| 1 | 6 | 1 | | 9 | 3 | 9 | 9 |
| 7 | 4 | 0 | 2 | | 0 | | 26 |
| 0 | 2 | 0 | 2 | 9 | 5 | | -3 |
| 1 | 8 | 1 | 2 | | 5 | 10 | 10 |
| | 7 | 0 | 1 | | | | 33 |
| 1 | 8 | 1 | | 2 | 8 | 16 | 16 |
| 1 | 1 | 0 | | 1 | 9 | | |
| 7 | 3 | 1 | | 9 | 12 | 12 | 12 |
| | 8 | 0 | 1 | | | | 17 |
| 1 | 3 | 0 | 8 | 8 | 15 | | 39 |
| 1 | 8 | 1 | 7 | | 10 | -6 | -6 |
| 1 | 1 | 0 | | 3 | | | 0 |
| 6 | 8 | 0 | 5 | | | | 13 |
| 1 | 1 | 0 | 2 | | 15 | | 3 |
| | 2 | 0 | 8 | | | | 7 |
| | 7 | 0 | 3 | | | | 5 |
| 1 | 7 | 0 | 2 | 6 | 7 | | 20 |
| 1 | 2 | 1 | | 6 | -2 | -13 | -13 |
| 1 | 4 | 1 | | 6 | 10 | 22 | 22 |

| cofirstto8w | cofirstto8lt | newcopdris | hypocopdclinic | summotivatrs2 | summotivatrs1 | newcopdsc |
|---|---|---|---|---|---|---|
| 28 | 28 | | | 16 | 15 | |
| | 11 | | | 25 | 20 | |
| | 11 | | | 16 | 16 | |
| | 0 | | | 17 | 10 | |
| 16 | 16 | | | 25 | 30 | |
| 43 | 42 | | | 11 | 22 | |
| | 5 | | | 24 | 27 | |
| | 8 | | | 25 | 28 | |
| 3 | 3 | | | 5 | 9 | |
| | 2 | | | 26 | 11 | |
| 20 | 20 | | | | 25 | |
| | 12 | | | 18 | 17 | |
| | 0 | | | 3 | 8 | |
| 43 | 36 | | | 30 | 26 | |
| | 1 | | | 16 | 28 | |
| | 17 | | | 18 | 36 | |
| 37 | 39 | | | 25 | 33 | |
| | | | | 18 | 16 | |
| 5 | 5 | | | 15 | 15 | |
| 30 | 30 | | | 11 | 11 | |
| | 1 | | | 20 | 33 | |
| 38 | 38 | 11 | 11 | 31 | 34 | 11 |
| | 7 | 9 | 9 | 29 | 33 | 9 |
| 15 | 15 | 7 | 7 | 41 | 13 | 7 |
| | 9 | 11 | 11 | 19 | 10 | 11 |
| | 15 | 10 | 10 | 29 | 38 | 10 |
| 18 | 18 | 9 | 13 | 23 | 29 | 9 |
| 8 | 8 | 8 | 8 | 25 | 22 | 8 |
| 18 | 18 | 4 | 8 | 32 | 24 | 4 |
| 6 | 6 | 4 | 8 | 27 | 24 | 4 |
| 34 | 34 | 12 | 12 | 28 | 35 | 12 |
| 11 | 11 | 4 | 4 | 24 | 31 | 4 |
| 10 | 10 | 10 | 10 | 32 | 38 | 10 |
| | 0 | 3 | 7 | 13 | 18 | 3 |
| 20 | 20 | 3 | 7 | 26 | 18 | 3 |
| 13 | 13 | 6 | 10 | 27 | | 6 |
| | 22 | 5 | 5 | 28 | | 5 |
| 18 | 18 | 11 | 11 | 24 | 20 | 11 |
| 42 | 42 | 7 | 11 | 19 | 17 | 7 |
| | 48 | 11 | 11 | 19 | | 11 |
| 4 | 4 | 12 | 12 | 23 | 32 | 12 |
| | 5 | 6 | 6 | 11 | 20 | 6 |
| | 2 | 6 | 10 | 28 | | 6 |
| 22 | 22 | 2 | 6 | 20 | 15 | 2 |
| | 20 | 1 | 5 | 26 | 37 | 1 |
| 14 | 14 | 2 | 6 | 31 | 24 | 2 |
| | 0 | 1 | 5 | 12 | 16 | 1 |
| 29 | 29 | 4 | 8 | 24 | 50 | 4 |

| | | | | | | |
|---|---|---|---|---|---|---|
| | 21 | 1 | 5 | 32 | 22 | 1 |
| | 26 | 1 | 5 | 24 | 23 | 1 |
| | -3 | 1 | 5 | 26 | 36 | 1 |
| 29 | 29 | 3 | 7 | 30 | 25 | 3 |
| 33 | 33 | 1 | 5 | | 37 | 1 |
| 40 | 40 | 6 | 6 | 24 | 26 | 6 |
| | | 1 | 5 | 9 | 17 | 1 |
| | 20 | 1 | 5 | 28 | 24 | 1 |
| 17 | 17 | 1 | 5 | | 12 | 1 |
| | 39 | 1 | 5 | 25 | 31 | 1 |
| 6 | 6 | 2 | 6 | 23 | 19 | 2 |
| | 0 | 6 | 6 | 20 | 18 | 6 |
| 13 | 13 | 1 | 5 | 23 | 24 | 1 |
| | 3 | 1 | 5 | 26 | 23 | 1 |
| | 7 | 3 | 7 | 27 | | 3 |
| | 5 | 1 | 5 | 35 | 30 | 1 |
| 20 | 20 | 1 | 1 | 19 | 18 | 1 |
| | -2 | 4 | 8 | 21 | 13 | 4 |
| | 28 | 1 | 5 | 33 | 25 | 1 |

| oldrespir | oldlifetime | newcopdlife |
| --- | --- | --- |
| 11 | 60 | 60 |
| 9 | 50 | 50 |
| 7 | 50 | 50 |
| 7 | 50 | 50 |
| 6 | 50 | 60 |
| 9 | 50 | 50 |
| 4 | 47 | 50 |
| 4 | 15 | 15 |
| 4 | 40 | 40 |
| 8 | 60 | 65 |
| 4 | 40 | 40 |
| 10 | 50 | 50 |
| 3 | 37 | 37 |
| 3 | 44 | 44 |
| 6 | 55 | 55 |
| 5 | 43 | 43 |
| 7 | 50 | 65 |
| 7 | 54 | 54 |
| 7 | 60 | 65 |
| 8 | 50 | 65 |
| 2 | 15 | 45 |
| 6 | 60 | 60 |
| 2 | 30 | 30 |
| 1 | 19 | 19 |
| 2 | 23 | 23 |
| 1 | 24 | 24 |
| 4 | 50 | 50 |

| | | |
|---|---|---|
| 1 | 22 | 22 |
| 1 | 19 | 19 |
| 1 | 15 | 15 |
| 3 | 31 | 31 |
| 1 | 14 | 14 |
| 2 | 29 | 47 |
| 1 | 18 | 18 |
| 1 | 27 | 27 |
| 1 | 22 | 22 |
| 1 | 16 | 16 |
| 2 | 38 | 38 |
| 2 | 35 | 47 |
| 1 | 17 | 17 |
| 1 | 32 | 32 |
| 3 | 43 | 43 |
| 1 | 18 | 18 |
| 1 | 32 | 32 |
| 4 | 40 | 40 |
| 1 | 19 | 19 |
